# Combining the AKT inhibitor capivasertib and SERD fulvestrant is effective in palbociclib-resistant ER+ breast cancer preclinical models

**DOI:** 10.1038/s41523-023-00571-w

**Published:** 2023-08-05

**Authors:** Lorna Hopcroft, Eleanor M. Wigmore, Stuart C. Williamson, Susana Ros, Cath Eberlein, Jennifer I. Moss, Jelena Urosevic, Larissa S. Carnevalli, Sara Talbot, Lauren Bradshaw, Catherine Blaker, Sreeharsha Gunda, Venetia Owenson, Scott Hoffmann, Daniel Sutton, Stewart Jones, Richard J. A. Goodwin, Brandon S. Willis, Claire Rooney, Elza C. de Bruin, Simon T. Barry

**Affiliations:** 1 Bioscience Early Oncology, AstraZeneca, Cambridge, UK; 2 Early Data Science, Oncology Data Science, AstraZeneca, Cambridge, UK; 3 Information Technology, AstraZeneca, Chennai, India; 4 Imaging Sciences, AstraZeneca, Cambridge, UK; 5 Bioscience Early Oncology, AstraZeneca, Boston, USA; 6 Translational Medicine, AstraZeneca, Cambridge, UK

**Keywords:** Breast cancer, Preclinical research

## Abstract

Combining the selective AKT inhibitor, capivasertib, and SERD, fulvestrant improved PFS in a Phase III clinical trial (CAPItello-291), treating HR+ breast cancer patients following aromatase inhibitors, with or without CDK4/6 inhibitors. However, clinical data suggests CDK4/6 treatment may reduce response to subsequent monotherapy endocrine treatment. To support understanding of trials such as CAPItello-291 and gain insight into this emerging population of patients, we explored how CDK4/6 inhibitor treatment influences ER+ breast tumour cell function and response to fulvestrant and capivasertib after CDK4/6 inhibitor treatment. In RB+, RB− T47D and MCF7 palbociclib-resistant cells ER pathway ER and Greb-1 expression were reduced versus naïve cells. PI3K-AKT pathway activation was also modified in RB+ cells, with capivasertib less effective at reducing pS6 in RB+ cells compared to parental cells. Expression profiling of parental versus palbociclib-resistant cells confirmed capivasertib, fulvestrant and the combination differentially impacted gene expression modulation in resistant cells, with different responses seen in T47D and MCF7 cells. Fulvestrant inhibition of ER-dependent genes was reduced. In resistant cells, the combination was less effective at reducing cell cycle genes, but a consistent reduction in cell fraction in S-phase was observed in naïve and resistant cells. Despite modified signalling responses, both RB+ and RB− resistant cells responded to combination treatment despite some reduction in relative efficacy and was effective in vivo in palbociclib-resistant PDX models. Collectively these findings demonstrate that simultaneous inhibition of AKT and ER signalling can be effective in models representing palbociclib resistance despite changes in pathway dependency.

## Introduction

Standard-of-care treatments for ER+ breast cancer (BC) commonly involve antagonising oestrogen receptor (ER) signalling using aromatase inhibitors or ERα antagonists^1,2^, ERα degraders (selective oestrogen receptor degraders (SERDs)) such as fulvestrant^3^ and more recently novel SERD molecules^4–6^. While targeting ER signalling delays tumour progression, tumour cells can escape ER inhibition through a cyclin-dependent kinase (CDK) 4 and 6 dependent mechanism^7–9^. The addition of CDK4/6 inhibitors to endocrine therapy increases therapeutic benefit in ER+ tumours and is now a commonly used treatment^10–12^.

ER+BC is also characterised by a high incidence of AKT pathway mutations, including common mutations in the PI3Kα sub-unit^13–16^. Alterations in PI3K-AKT signalling (through mutation or pathway activation) drive tumour progression together with other signalling drivers such as ER. Moreover, the PI3K-AKT and ER signalling pathways have the potential to cross-talk, with upregulated PI3K signalling driving endocrine resistance^17^ and ER upregulation^18^. The addition of the PI3Kα inhibitor alpelisib to fulvestrant has demonstrated efficacy in CDK4/6 inhibitor naïve PI3Kα mutant tumours, with a very limited number of patients receiving prior CDK4/6 treatment included in the SOLAR-1 Phase III trial^19^. Moreover, prior to the introduction of CDK4/6 inhibitors, the mTORC1 inhibitor everolimus, in combination with aromatase inhibitors, improved PFS in an unselected patient population^20^. More recently, a number of clinical studies in which monotherapy fulvestrant treatment has been given after CDK4/6 inhibition in combination with exemestane have shown a shorter progression-free survival in comparison with studies conducted prior to the introduction of the CDK4/6 inhibitors^21,22^ suggesting combinatorial approaches are needed for the post-CDK4/6 inhibitor population.

Collectively preclinical and clinical data demonstrate that combined targeting of either ER inhibitors with CDK4/6 inhibitors (palbociclib, abemaciclib or ribociclib) or ER inhibitors with inhibitors of PI3K, mTORC1 or AKT gives benefit in patients with metastatic ER+BC. Studies exploring resistance mechanisms to combined ER and CDK4/6 inhibitor treatment reveal that cross-talk or emerging resistance is associated with increased PI3K-AKT pathway signalling. For example, resistance to ribociclib and the aromatase inhibitor letrozole is associated with loss of PTEN, resulting in activation of PI3K-AKT signalling^23^. Notably, loss of PTEN also mediates resistance to alpelisib, further emphasising the importance of PI3K-AKT signalling^23–25^. Finally, deletion of PDK1 prevented resistance to CDK4/6 inhibitors in ER+ breast cancer cell lines ^26^.

The selective AKT1,2,3 inhibitor capivasertib (AZD5363)^27^ has shown statistically significant and clinically meaningful improvement in PFS in the Phase III trial CAPItello-291 in ER+BC when combined with fulvestrant^28^. With the increased use of CDK4/6 inhibitors in combination with aromatase inhibitors in early-stage BC, patients with advanced BC are more likely to have tumours that have progressed following treatment with a CDK4/6 inhibitor. Reflecting this, CAPItello-291 enrolled patients who received prior endocrine therapy with or without CDK4/6 inhibitors, with the majority of randomised patients receiving prior CDK4/6 inhibitor treatment. While a small uncontrolled single-arm trial (BYLieve) suggested that combined alpelisib and fulvestrant treatment has some activity in the post CDK4/6 setting^29^, the impact of CDK4/6 inhibitor treatment on PI3K-AKT pathway function alone or in combination is poorly understood. To explore the influence on response to ER and AKT antagonists, we have used a panel of CDK4/6 inhibitor-resistant cell lines and PDX models to study the response to capivasertib and fulvestrant monotherapy activity and combination treatment.

## Results

### Palbociclib-resistant cell lines associated with RB loss vs RB retention have distinct signalling and transcriptional profiles

**Fig. 1:**
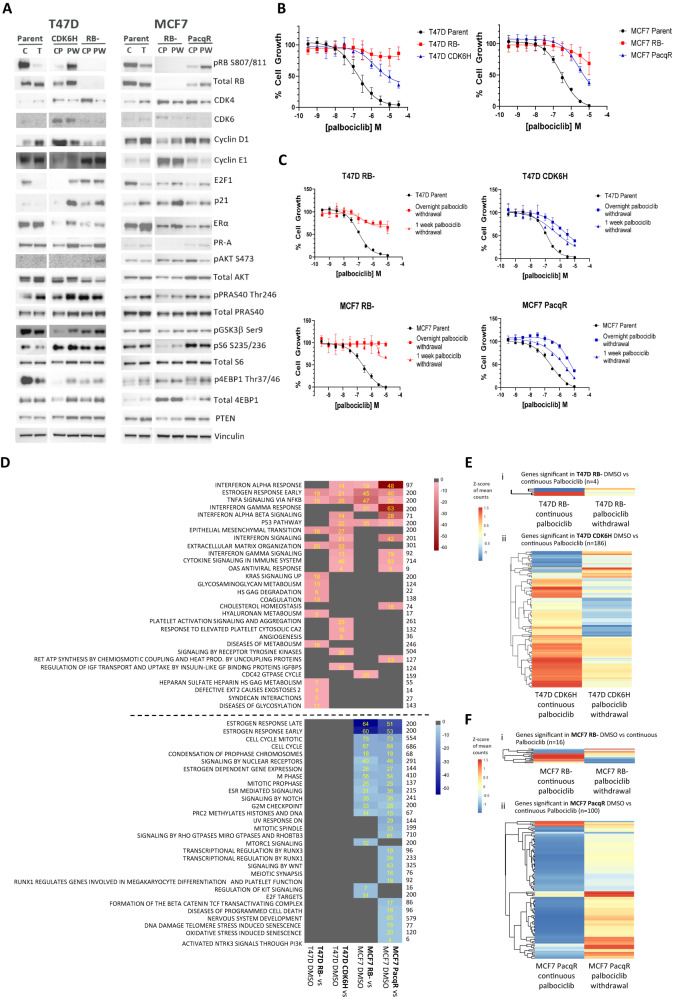
RB− and RB+ palbociclib-resistant cell lines have distinct signalling and transcriptional profiles. **A** Western blot profiling of PalboR cells for cell cycle, ER-regulated and PI3K pathway markers. Cells cultured long-term with continuous palbociclib (CP) 3 μM T47D, 1 μM MCF7, palbocilib withdrawn (PW) for 1 (MCF7) or 2 (T47D) weeks. Parental cells treated for 24 h with DMSO (C) or palbociclib (T) 3 μM T47D, 1 μM MCF7. **B** Effect of palbociclib on cell proliferation in parental versus PalboR cells plated without palbociclib overnight and then treated for 5 days with palbociclib at concentrations indicated. Mean ± SD of three independent experiments performed with duplicates represented. **C** Stability of resistance phenotype measured by removing palbociclib for 1 and 7 days, then retreating palbociclib for 5 days. Mean duplicate cell counts ± SD representative of three independent experiments. **D** PalboR cells in continuous palbociclib compared to parental cell lines treated with DMSO. Pathway heatmaps of the top 30 pathways ordered by combined log *p*-value across treatment groups as indicated; upregulated pathways red (top heatmap), downregulated pathways blue (bottom heatmap). Shade represents log *p*-value. Numbers to the right of the heatmap represent the total number of genes in the pathway signature, yellow numbers in boxes DEG found in signature. RET respiratory electron transport, PROD production, GF growth factor. **E**, **F** Heatmap showing **E** T47D and **F** MCF7 mRNA *z*-scores of (i) differentially expressed genes in T47D RB− and MCF7 RB− following palbociclib withdrawal compared to continuous treatment, (ii) differentially expressed genes in T47D CDK6H and MCF7 PacqR following palbociclib withdrawal compared to continuous treatment. Each row represents a gene with significant differential expression between groups in one or both resistant cells (−1 > log2FC > 1 and *p*-value (FDR) < 0.01). No genes were found in common between (i) and (ii) for T47D or MCF7-resistant cell lines.

To investigate how palbociclib treatment may influence ER+BC cells, a panel of palbociclib-resistant cell pools was generated. MCF7 (PIK3CA E545K mutant) and T47D (PI3KCA H1047R mutant) cells were used. Treatment of parental T47D and MCF7 with palbociclib-modified biomarkers associated with cell cycle progression consistent with the mode of action and reduced cell growth (Fig. 1A, B). Continuous palbociclib treatment (CP) generated two resistant (PalboR) cell pools (T47D RB− and MCF7 RB−) with RB deletion, which was stable upon palbociclib withdrawal (PW) (Fig.1A; Supplementary Fig. 1A). A second T47D pool (T47D CDK6H) had increased CDK6 expression (Fig. 1A), which remained stable on drug withdrawal. The second MCF7 PalboR pool (MCF7 PacqR) had downregulation of RB and E2F1 protein (but not genetic loss) and upregulation of CDK4 protein expression on continual drug exposure, which partially rebounded on drug withdrawal (Fig. 1A). The changes observed mirror those observed in patients post-palbociclib treatment^30^. All PalboR cells showed reduced growth following palbociclib treatment; T47D RB− & MCF7 RB− cells were most resistant, with sensitivity reduced 10-fold in T47D CDK6H & MCF7 PacqR cells (Fig. 1B). Importantly resistance to palbociclib partially reversed in RB+ cells over time, while RB− did not change following palbociclib removal.

Both the MCF7 and T47D-derived PalboR cells had lower expression of ERα (Fig. 1A) consistent with other studies^31^, suggesting changes in ERα signalling or function post-palbociclib exposure. In addition, the MCF7 RB− pool had reduced PR (Fig. 1A) and GREB1 protein expression (Supplementary Fig. 1B). Palbociclib withdrawal (PW) resulted in a partial rebound of ERα expression in some cells. There were also changes in the PI3K/AKT pathway activation status in the MCF7 PacqR, T47D CDK6H and T47D RB− resistant cells, with marked upregulation of pS6 (Fig. 1A), while pS6 was reduced in the MCF7 RB−. Interestingly downregulation of TSC1 and TSC2, which regulates p-70S6K and mTORC1 mediated modulation of pS6, was seen in the MCF7 PacqR cells. (Supplementary Fig. 1B). Upon palbociclib removal, only T47D CDK6H consistently retained sustained activation of pS6. In other cells, sustained pS6 was variable between experiments suggesting a non-genetic activation. Following palbociclib withdrawal (PW) from the PalboR cells T47D RB− and MCF7 RB− cells, there was no change in sensitivity to subsequent re-treatment with palbociclib (Fig. 1C). In T47D CDK6H and MCF7 PacqR cells, which retain some (albeit lower versus parental cells) degree of sensitivity to palbociclib in culture, resistance was partially reversed following palbociclib removal (Fig. 1C).

To explore the consequence of long-term CDK4/6i exposure, the gene expression profiles of PalboR cells grown continuously on palbociclib were compared to the parental cells cultured with DMSO. (Fig. 1D, Supplementary Fig. 1C, D). Significant differentially expressed genes between the parental and PalboR cells are shown as a heatmap of the *z*-scores to indicate relative directional changes. The expression changes in the RB− and RB+ PalboR cells relative to the parental cells were different across the resistant cell pools (Supplementary Fig. 1C, D). Pathway enrichment (or over-representation analysis) was applied to explore the enrichment of downregulated and upregulated differentially expressed genes in hallmark^32^ and reactome pathways (taken from the MSigDB^33^). Whilst T47D PalboR cell lines had only upregulated pathways after continuous palbociclib, in MCF7 PalboR cell lines, a number of pathways were downregulated. Oestrogen response early and TNFα (tumour necrosis factor α) signalling via NFKB were found to be upregulated across all PalboR cells. Interestingly upregulation of specific ER genes such as *TFF1* and *AREG* associated with ER response contrasts with the downregulation of ER protein expression in resistant pools. Furthermore, upregulation and downregulation of ER-responsive genes in MCF-7 may indicate that rather than simple upregulation of ER signalling, there is some overlap with other pathways (Supplementary Table 1). Within TNF signature, a number of the genes significantly modulated are related to cell signalling and survival pathways (Supplementary Table 2). T47D RB− cells also had changes in metabolic pathway gene expression, while interferon signalling regulated genes were modified in the CDK6H cells as shown in other studies^34^. MCF7 PalboR cells had greater pathway overlap, including oestrogen response, interferon response, p53 and cell cycle pathways (Fig. 1D).

The impact of removing palbociclib on gene expression was also examined. The RB− cells gene expression profiles were stable, with only 4 and 16 differential expressed genes changing on palbociclib removal in T47D and MCF7 RB−, respectively (Fig. 1Ei, Fi). The same genes were not modulated following palbociclib removal in RB+ cells (Fig. 1E, F). In contrast, 186 and 100 genes were found to be differentially expressed in T47D CDK6H and MCF7 PacqR, respectively, following palbociclib removal (Fig. 1Eii, Fii). This suggests that resistance in the T47D CDK6H and MCF7 PacqR cells may be adaptive and not driven by one particular change. Collectively, the data implies RB genetic loss develops an intrinsic resistance profile, while in RB+ cells, changes in expression of cell cycle regulating proteins and other signalling pathways are adaptive with reduced palbociclib resistance which is not maintained when CDK4/6 inhibitor is withdrawn.

### Chronic palbociclib exposure impacts both PI3K pathway and ER pathway inhibition

**Fig. 2:**
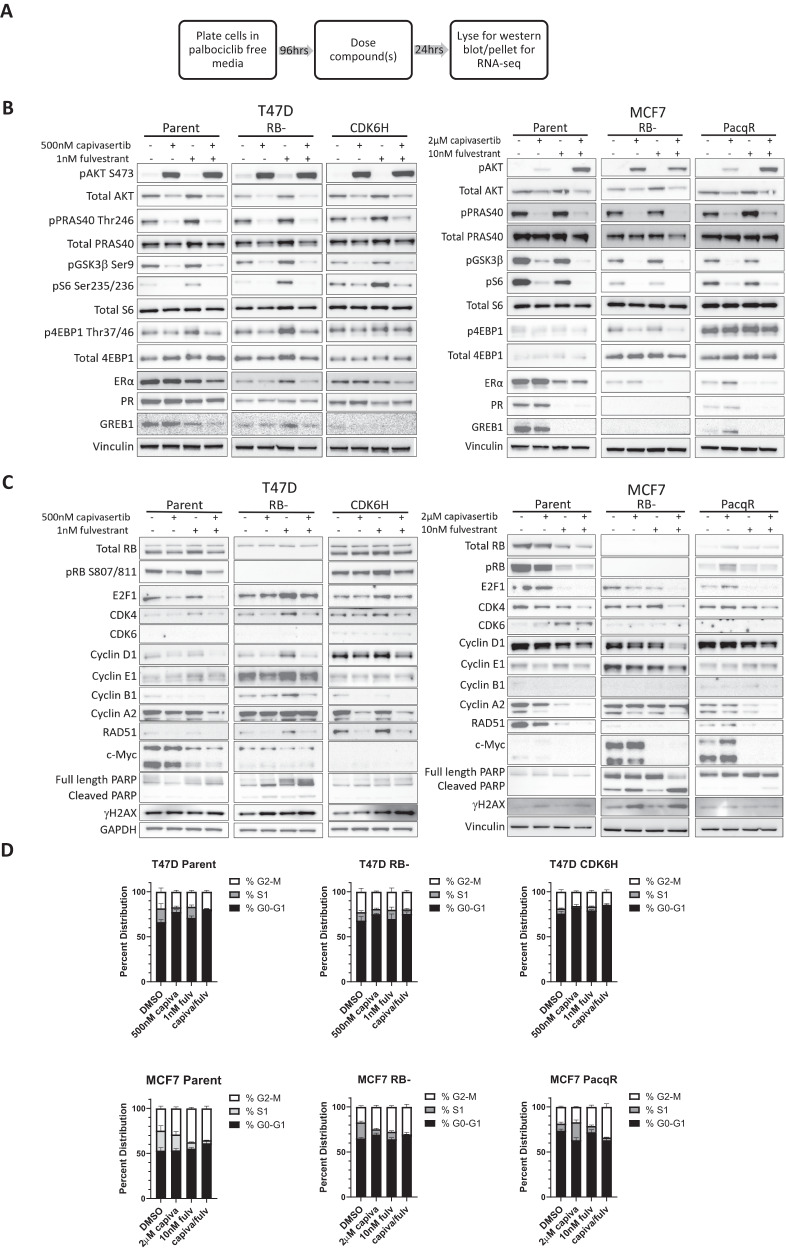
Palbociclib exposure impacts PI3K pathway, ER pathway and cell cycle response. **A** Treatment schedule. **B** Analysis of PI3K/AKT and ER pathway biomarkers and **C** cell cycle and apoptosis biomarkers of parental and PalboR T47D & MCF7 cells plated in the absence of palbociclib for 96 h prior to treatment with capivasertib, fulvestrant and the combination for 24 h at the doses indicated. Representative example of two independent experiments. **D** Cell cycle analysis of the EdU stained parental and PalboR T47D & MCF7 cells following palbociclib removal for 96 h prior to a 48-h treatment with capivasertib, fulvestrant and the combination at the doses indicated. Mean cell distribution of each cell cycle phase plotted ± SD (*n* = 4 biological replicates).

To understand if long-term palbociclib exposure changes signalling output from the PI3K and ER pathways, cell function and drug response, parental and PalboR cells were treated with monotherapy capivasertib and fulvestrant, and the combination (Fig. 2A). PalboR cells were cultured in the absence of palbociclib prior to addition of capivasertib, fulvestrant or the combination to remove any potential direct influence of palbociclib. Consistent with the capivasertib mode of action AKT phosphorylation was induced in treated cells, and the PI3K-AKT pathway biomarkers PRAS40 and GSK3β were inhibited in all cell lines. The downstream PI3K-AKT biomarker pS6 was similar to parental cells in the T47D RB−, MCF7 RB− and MCF7 PacqR cells, but upregulated in T47D CDK6H suggesting dysregulation of pathway below or by factors in addition to AKT (Figs. 1A and 2B). Following treatment with monotherapy capivasertib and the combination, the decrease in pS6 S235/S236 levels was less pronounced in T47D CDK6H and MCF7 PacqR cells versus parental and both RB− cells at the concentrations used (Fig. 2B). This suggests that palbociclib resistance in the RB+ resistant cells could be influenced by increased activation of, or differential signalling through, the PI3K-AKT-mTOR pathway. Other studies have shown that both AKT, PI3K^35^ or mTORC1^36^ can be important in palbociclib-resistant cells. The ER axis also showed a difference in activation status in parental versus PalboR cells. Downregulation of ER expression occurred in all four PalboR cell lines as well as reduced expression of GREB1 (ER pathway output biomarker), possibly indicating reduced output and dependency on ER signalling in the PalboR cell lines.

The direct impact of the monotherapy and combination treatment on cell cycle biomarkers was also investigated. The combination downregulated E2F1 and Cyclin D1 in both parental T47D and MCF7 cells (Fig. 2C); this was attenuated in the T47D PalboR cells but not the MCF7 PalboR cells. Notably, there was differential modulation of Myc across the cell lines. In the T47D parental cells, Myc was moderately reduced by fulvestrant and the combination and less so in the T47D RB−. Fulvestrant and the combination treatment markedly reduced expression in the MCF7 RB− and PacqR cells. In MCF7 parental and T47D CDK6H, Myc was barely detectable. An increase in cleaved PARP and γH2AX was seen with capivasertib and in combination in both RB− cells (Fig. 2C). Each monotherapy treatment had differential effects on the cell cycle (Fig. 2D, Supplementary Fig. 2A). The individual impact of capivasertib and fulvestrant monotherapy varied across the parental and resistant lines, but the combination treatment generally resulted in a reduction of the fraction of cells in S-Phase and an increase in G1-M (Fig. 2D, Supplementary Fig. 2A–C). The impact of the monotherapy and combination treatment on other phases of the cell cycle differed between the MCF7 and T47D parental as well as the T47D and MCF7 PalboR resistant cells; this was most marked in the MCF7 PacqR cells (Supplementary Fig. 2B, C). Collectively these data suggest that despite dysregulation of cell cycle control upon palbociclib resistance, the combination of capivasertib and fulvestrant still impacts cell cycle progression. However, importantly the profile of biomarkers at baseline and the changes in response to each treatment are heterogenous, with differences seen between all the cells.

### Transcriptional changes in response to capivasertib, fulvestrant or the combination treatment are attenuated in PalboR cells

To gain greater insight into the impact of capivasertib, fulvestrant and the combination, transcriptomic analysis of the T47D and MCF7 cells was performed (detailed summary outlined in Supplementary Results and Supplementary Figs. 3, 4 and 5). In T47D parental cells, capivasertib modulated E2F signalling, cell cycle regulation and DNA repair, while fulvestrant downregulated genes associated with ER signalling (Supplementary Fig. 3B–D). The combination induced deeper and more consistent change in genes modulated by monotherapy capivasertib and a modest improvement in the modulation of fulvestrant-regulated genes (Supplementary Fig. 3A–D) plus an additional 21 downregulated pathways (Supplementary Fig. 3E). This suggests targeting AKT and ER in T47D is complementary. In the MCF7 parental cells, capivasertib modulated far fewer genes, while fulvestrant modulated E2F, cell cycle and ER pathway-associated genes. The combination further increased the modulation of the fulvestrant-regulated genes (Supplementary Fig. 4A–E).

**Fig. 3:**
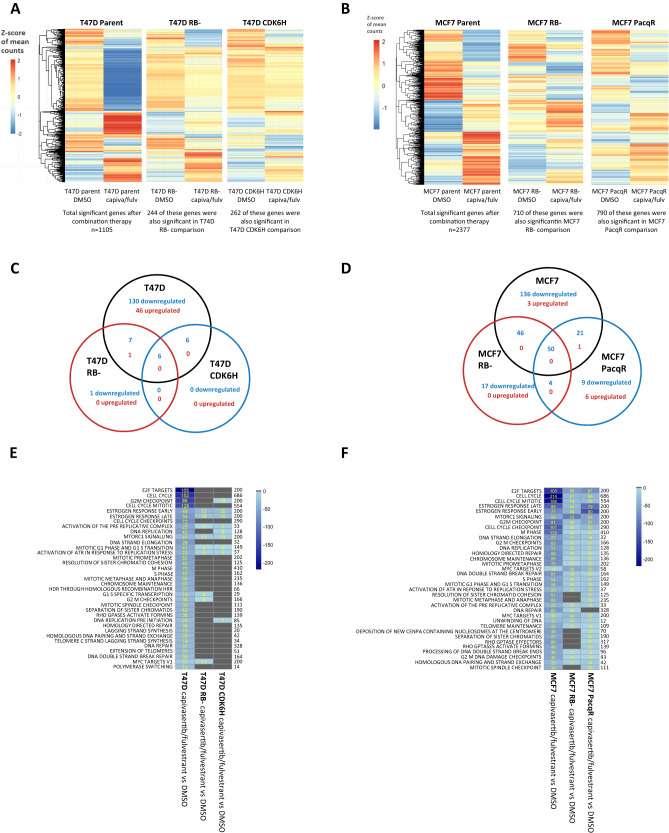
Transcriptomic profile of the capivasertib fulvestrant combination in parental versus palbociclib-resistant cells. **A** Significant differentially expressed genes after combination treatment in parental T47D (*z*-scores of the mean expression). Each row represents a single gene. Columns represent relative expression for DMSO and combination treatment in parental, T47D RB−, T47D CDK6H cells. The number of genes that were significantly differentially expressed in PalboR lines as well as parental for combination vs DMSO are shown as the common genes below each column (the total number of DEG found in T47D RB− was 411 and for T47D CDK6H 484). **B** Significant differentially expressed genes after combination treatment in parental MCF7 are shown as *z*-scores, with each row representing a single gene with significant differential expression. Columns representing the changes in the same genes are shown for DMSO and capivasertib fulvestrant combination treatment comparing parental, MCF7 RB−, MCF7 PacqR cells. The number of genes that were also found to be significantly differentially expressed in PalboR lines are shown as the common genes below the relevant columns. MCF7 RB− had a total of 1531 DEG in comparison of capivasertib fulvestrant vs DMSO and MCF7 PacqR had a total 2300 DEG. **C**, **D** Overlap of downregulated genes enriched in pathways (blue) and upregulated genes enriched in pathways (red) in the comparison of combination treatment with DMSO in **C** T47D parental, T47D RB−, T47D CDK6H cells and **D** MCF7 parental, MCF7 RB− and MCF7 PacqR. **E**, **F** Pathway heatmaps representing the top 35 pathways ordered by combined log *p*-value across the groups. Pathways that are enriched in the downregulated DEG are shown in blue. All comparisons are combination vs DMSO (control) in each of **E** the three T47D cell lines and **F** the three MCF7 cell lines. Shade represents log *p*-value, the numbers to the right of the heatmap the total number of genes in the pathway and yellow numbers in boxes the DEG found in the pathway.

To assess the relative changes in gene expression following compound treatment in parental versus PalboR cells, palbociclib was removed for 96 h, and PalboR cells were then treated with capivasertib, fulvestrant or the combination. The effect of monotherapy and combination treatment on the differentially expressed genes associated with response in parental cell lines was examined. In the combination-treated PalboR cells, the changes in the combined panel of treatment-responsive genes defined in the parental cell lines were attenuated (Fig. 3A, B). A similar effect was seen in the monotherapy treatments (Supplementary Fig. 5A–D). These relative differences in gene expression suggest that the genes modified by capivasertib monotherapy in parental cells do not show the same response in the PalboR cells. This suggests there is a difference in the impact of capivasertib treatment on transcriptional profiles in the context of CDK4/6i resistance. In addition, the data suggest that the downregulation of ER signalling following fulvestrant treatment may also be reduced in the PalboR cells.

To independently compare the response of parental and PalboR cells to the combination treatment, the differentially expressed genes following treatment of each individual cell line were determined. This revealed a marked reduction in the numbers of genes associated with cell cycle regulation in both T47D RB− and T47D CDK6H cells compared to the parental cells (Fig. 3E). Regulation of genes associated with oestrogen pathway modulation was still observed, although the degree of downregulation was lower than in the parental cells (Fig. 3C, E). Although attenuation of gene expression also occurred in the MCF7 parental versus MCF7 PalboR cells, the reduction in expression was less marked (Fig. 3D, F). Interestingly despite high Myc expression in MCF7 PalboR cells, a reduction in Myc target genes was still apparent consistent with a reduction of Myc protein expression (Fig. 2C). Collectively the gene expression analysis revealed that the capivasertib fulvestrant combination modulates cell cycle, Myc and ER target genes in T47D RB−, MCF7 RB− and MCF7 PacqR cells whereas DNA replication, cholesterol biosynthesis-homoeostasis and ER genes were modulated in the T47D CDK6H cells. The overlapping pathways are shown in Supplementary Tables 3–11. The diverse transcriptional response to the capivasertib/fulvestrant combination suggests the four PalboR cells have distinct resistance mechanisms, and palbociclib resistance results in changes in the transcriptional responses to both AKT and ER targeting.

### Palbociclib-resistant cells retain sensitivity to the combination of capivasertib and fulvestrant

**Fig. 4:**
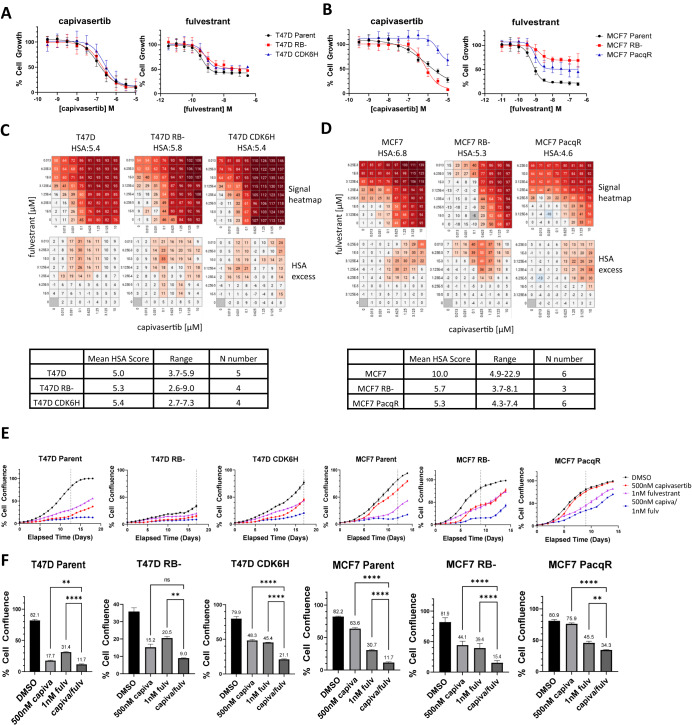
Sensitivity of palbociclib-resistant cells to the combination of capivasertib and fulvestrant. **A** T47D and **B** MCF7 parental and PalboR cells plated without palbociclib overnight, then treated with capivasertib and fulvestrant at concentrations indicated for 5 days. Cell count normalised to DMSO control (100%) and cell count at the time of dosing (0%). Mean of **A** 4 and **B** 3 independent experiments. **C** T47D and **D** MCF7 parental and PalboR cells treated with capivasertib and fulvestrant at concentrations indicated for 5 days. Normalised cell counts analysed by Genedata Screener Compound Synergy Extension. Representative combination clusters are shown (0 represents DMSO control, values 1–100 represent cell growth inhibition, values > 100 represent cell death) with Highest Single Agent (HSA) excess heatmaps (highlights dose range where combination benefit was detected) from representative experiment. Tables summarise the range of HSA scores for 3+ independent experiments. **E** Parental and PalboR T47D and MCF7 cell confluence measured by Incucyte S3. Cells treated 4 days on 3 days off 500 nM capivasertib, continuous 1 nM fulvestrant, or combination for ≥14 days as indicated. **F** Relative growth inhibition following monotherapy and combination treatment. Confluence calculated at day 18 or 80% control confluence (time point represented by dotted vertical lines on (**E**). Representative data of ≥3 independent experiments shown with ordinary 1-way ANOVA (Analysis of Variance) statistical analysis *p < 0.05; **p < 0.01; ***p < 0.005, ****p < 0.001.

The PalboR cell lines were then used to assess how palbociclib resistance influences sensitivity to capivasertib, fulvestrant and the combination. As experiments were performed in the absence of palbociclib, the T47D CDK6H and the MCF7 PacqR cells will have partially reverted (Fig. 1). In a 5-day proliferation assay the activity of capivasertib (intermittent treatment 4 days on 3 days off mimicking the clinical schedule) and fulvestrant was similar in the T47D RB− & CDK6H cells versus parental T47D (Fig. 4A). Capivasertib was marginally more effective in the MCF7 RB− cells versus parental cells, but less effective in MCF7 PacqR cells (Fig. 4B). Fulvestrant activity was reduced in both the MCF7 RB− & MCF7 PacqR cells relative to parental MCF7 (Fig. 4B). To examine the changes in combination benefit a combination matrix screen was performed. Synergy scores indicated that combination benefit is maintained in T47D and reduced in the MCF7 PalboR cells (Fig. 4C, D). Despite this, the combination gave the greatest anti-proliferative activity in both parental and PalboR cells. In the PalboR T47D cells, the combination benefit was equivalent to parental cells (Fig. 4C).

To test the durability of response, a long-term growth assay was performed using the 4 days on 3 days off capivasertib schedule. Although MCF7 PalboR cells were sensitive to the combination, at 500 nM capivasertib maximal growth inhibition was reduced compared to the parental cells, consistent with reduced capivasertib and fulvestrant monotherapy activity in the MCF7 RB− cells and markedly reduced activity in MCF7 PacqR cells at the doses used (Fig. 4D). In all cells the combination was the most effective treatment (Fig. 4E, F). This is, in part, a result of using a 500 nM dose of capivasertib; when the concentration of capivasertib is increased to 2 μM greater added benefit is observed (data not shown), consistent with data in the combination screen (Fig. 2D). Collectively the data suggest the combination of capivasertib and fulvestrant has potential to reduce the growth of cells resistant to CDK4/6 inhibition through loss of RB− or modification of other pathways, although the degree of anti-proliferative effect, and additive benefit from combination treatment may be reduced.

### Anti-tumour efficacy of the capivasertib and fulvestrant combination in palbociclib insensitive PDX models

**Fig. 5:**
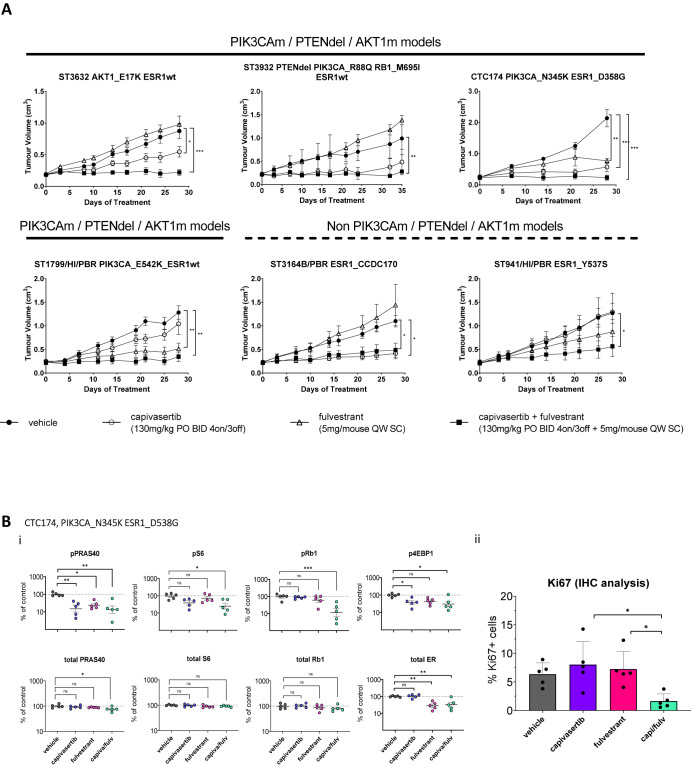
In vivo anti-tumour activity of the combination of capivasertib and fulvestrant in palbociclib insensitive models. **A** In vivo activity of capivasertib, fulvestrant and the combination in PI3K pathway mutated PDX tumour models (ST3632, ST3932, CTC174 and ST1799/HI/PBR) and PI3K pathway unaltered models ST3164B and ST941/HI/PBR. Tumours treated with vehicle (closed circles), 130 mg/kg capivasertib BID 4 days on 3 days off (open circles), 5 mg/animal fulvestrant, QW (open triangle), or combination (closed squares). Geomean tumour volumes ± SEM (*p < 0.05, **p < 0.005, ***p < 0.0005) are shown. **B** (i) Pharmacodynamic changes phosphorylation and total protein levels of PRAS40 (Thr246), S6 (Ser235/236) and p-4EBP1 (Thr37/46), pRb1 or ER in the CTC174 PDX PI3KCAm model after 28 days of treatment. Tumours treated with vehicle, 100 mg/kg capivasertib BID 4 days on 3 days off, 5 mg/animal fulvestrant, or combination. Data normalised to the geomean of β-actin and vinculin, percentage change from control plotted as mean ± SEM (*n* = 5). Statistical analysis ANOVA test vs vehicle-treated, *p < 0.05; **p < 0.005; ***p < 0.0005. (ii) Ki-67 was visualised using immunohistochemistry of tumour samples; represented as mean ± SEM (*n* = 5). Statistical analysis ANOVA test, *p < 0.05; **p < 0.01; ***p < 0.005, ****p < 0.001.

To confirm the combination benefit translates in vivo, the efficacy was tested in a panel of palbociclib non-responsive PDX models, which were insensitive or showed modest response to palbociclib at the 50 mg/kg or 25 mg/kg QD dose as indicated (Supplementary Fig. 6). The combination gave greatest anti-tumour activity in the six models that included PI3K-AKT pathway-altered and non-altered tumours (Fig. 5A). This included ST3932 tumours which in addition to being PTEN null, PIK3CA mutant also carried a RB1 mutation. Interestingly in the ST3164B/PBR model, which has no detected PI3K-AKT pathway alteration but carries an ESR1 fusion, fulvestrant was inactive; however, capivasertib treatment reduced growth, indicating potential for AKT dependency in the absence of pathway alteration. In addition, the combination showed the greatest activity in a broader ER+ breast PDX tumour panel screen (Supplementary Fig. 7A). Therefore, the combination of capivasertib and fulvestrant reduces tumour growth in tumours that are CDK4/6 inhibitor resistant or have been exposed to CDK4/6 inhibitor long term and has efficacy in tumours without mutations in the PI3K-AKT pathway.

The pharmacodynamic effects following treatment with capivasertib, fulvestrant and the combination were assessed in the ER-positive breast cancer PDX model CTC174 (PI3KCAm) (Fig. 5B). Each monotherapy treatment reduced relevant AKT pathway and ER biomarkers (Fig. 5Bi), while the combination reduced markers of both pathways which translated into greater impact on markers of the cell cycle (pRB1) (Fig. 5Bii) and proliferation (Ki67) (Fig. 5Bii). The dose response of capivasertib in combination with fulvestrant was further explored in the ST1799/HI/PBR model. Increasing monotherapy doses of capivasertib resulted in greater anti-tumour activity, with maximal tumour response seen in combination with fulvestrant (Supplementary Fig. 7B). Greatest modulation of Ki67 was observed at the 100 mg/kg and 130 mg/kg 4 days on 3 days off dose of capivasertib in combination with fulvestrant, mirroring the effect in the CTC174 model (Supplementary Fig. 7C). Collectively the data support a broad impact of capivasertib in combination with fulvestrant in tumour models with different mutational profiles.

## Discussion

Here we have investigated the specific impact of palbociclib resistance on the subsequent response to the combination of capivasertib and fulvestrant and each monotherapy. We show that CDK4/6 inhibitor resistance following long-term exposure to palbociclib has the potential to upregulate PI3K-AKT signalling, downregulate ER pathways, and influence subsequent effects of both AKT and ER inhibition on tumour cell signalling and function. Despite this, combined treatment with capivasertib and fulvestrant is still effective in cell lines and tumours that were resistant or non-responsive to palbociclib. However, the magnitude of activity may be reduced relative to the effect that can be achieved in palbociclib naïve cells.

There is now evidence that the clinical effectiveness of ER inhibition may be reduced following exposure to CDK4/6 inhibition^21,22^. In the PalboR cell lines, ER expression was reduced compared to parental cells, and in the MCF7 RB− cells, expression of the ER biomarker GREB1 was lost, consistent with a pattern of reduced pathway output. Although the ER pathway transcripts were modulated by fulvestrant alone and the combination, the degree of modulation was reduced in the PalboR cells. Gene expression analysis of T47D and the MCF7 palbociclib naive and PalboR cells showed fewer ER pathway genes were suppressed versus parental cells, though this was more marked in the T47D cells. Despite this, the combination consistently reduced the expression of the ER-dependent protein GREB-1. Other ER inhibitors, such as the SERD/SERM elacestrant, have shown activity in CDK4/6i resistant models^37^. The in vitro cell assays suggest that while fulvestrant still reduces cell growth, it may be less potent once CDK4/6 resistance develops. This could be due to either attenuation of ER dependency or activation of other pathways. The PI3K-AKT pathway was also modulated upon the acquisition of palbociclib resistance. pS6 was upregulated in the T47D CDK6H cells and was less effectively reduced in both T47D and MCF7 PalboR RB+ cells at the concentrations of capivasertib examined. This may indicate that in these cells, there is reduced sensitivity to AKT monotherapy or combination inhibition due to increased activation of the PI3K-AKT node or upregulation of mTORC1 signalling.

A number of studies have identified genetic alterations associated with CDK4/6 inhibitor resistance or tumour adaption in patient samples^30,31,38^, and by screening preclinical models^26,35,36,39–41^. Reduced sensitivity to CDK4/6 inhibitors is associated with alterations that impact cell cycle checkpoints, such as loss of RB1^42^, increased expression of CDK6^31,43^, expression of p16^41^, and ink6^39^. Other mechanisms of resistance are also evident, including FGFR activation^44^ and alterations in PTEN^23^. Tumours profiled upon progression following CDK4/6 and aromatase inhibitor treatment are enriched in alterations of the PI3K-AKT pathway^38^. This establishes PI3K, AKT or mTORC1/2 signalling plays an important role in tumour progression in the context of CDK4/6 inhibitor treatment. A number of preclinical studies have established proof of principle that combining mTORC1^35^ or PI3Kα^35^ inhibitors with ER antagonists gives anti-tumour activity in PIK3CA mutant tumours. One study^45^ showed AKT inhibition with capivasertib gave increased benefit in MCF-7 and T47D RB+ resistant cell lines resistant to both fulvestrant and CDK4/6 inhibitors^45^. Their data suggest that AKT inhibition in combination with fulvestrant had reduced activity relative to parental cells, albeit at different drug concentrations, and although the combination of capivasertib and fulvestrant was not tested in vivo, the ability of capivasertib to enhance the activity of fulvestrant and CDK4/6 inhibition was demonstrated. In our study, we specifically tested the impact of palbociclib resistance on response to the capivasertib fulvestrant combination. Our data shows anti-proliferative activity in both RB− and RB+ PalboR cells, where resistance was associated with different changes in tumour cell signalling status. Moreover, the combination of capivasertib and fulvestrant had activity in PDX models that were insensitive to palbociclib and with either no or modest response to fulvestrant. Consistent with the in vitro data, a combination benefit was observed in a PDX model harbouring an RB mutation. The activity of the combination was observed in PDX models representing PI3K-AKT pathway-altered and non-altered tumours. While the degree to which capivasertib or fulvestrant delivered respective anti-tumour benefit varied between models, the data exemplify how adding capivasertib to fulvestrant could show greater anti-tumour benefit in an overall population versus fulvestrant alone. Given the heterogeneous changes associated with CDK4/6 inhibitor treatment, it would be interesting to understand in more detail where the addition of a CDK4/6 inhibitor or maintenance of CDK4/6 inhibitor treatment in the post-CDK4/6 inhibitor population could give further benefit in the context of the capivasertib fulvestrant combination.

Prolonged palbociclib treatment is associated with effects on cell cycle regulation^7,39,42^. Transcript profiling of T47D RB−, T47D CDK6H, MCF7 RB− and MCF7 PacqR cells versus parental revealed diverse changes in gene expression changes following treatment, in particular, a reduction in the degree of modulation of genes associated with cell cycle following combination treatment. Despite this, the combination induced a similar pattern of cell cycle arrest across the parental and resistant cell lines. Similar potency was seen in proliferation assays versus parental cells for the T47D PalboR cells; reduced efficacy was seen in the MCF7 PalboR cells, where capivasertib is less effective at inhibiting proliferation. This may suggest that while CDK4/6 treatment tumours may show differential pathway activation and sensitivity to monotherapy treatments, combinations such as capivasertib and fulvestrant can be effective even without the same impact on cell cycle pathways, possibly as a result of targeting other critical cellular processes.

Based on this current study and other complementary studies^26,35,45^, it is apparent that in the context of a combinatorial treatment, capivasertib and fulvestrant contributions to efficacy may drive different effects. While the post-CDK4/6i tumours may be intrinsically less sensitive to treatment, consistent with the findings in CAPItello-291^28^ the combination of capivasertib and fulvestrant does, however, offer potential for a broader therapeutic effect than that achieved by fulvestrant alone.

## Methods

### Cell culture and reagents

All cell lines were authenticated using DNA fingerprinting short-tandem repeat (STR) assays. To generate resistant cell populations, parental cell lines were exposed to escalating concentrations of palbociclib over 4–6 months. T47D cells were cultured up to a final dose of 3 μM, which generated two PalboR cell pools (named T47D RB− & T47D CDK6H). MCF7 PalboR cell pools (named MCF7 RB− & MCF7 PacqR) were previously generated and named MCF7 PC8 & MCF7 PC6^36^, respectively, by continuous culture up to a final dose of 1 μM. Capivasertib, palbociclib and fulvestrant (AstraZeneca) were dissolved in dimethyl sulfoxide (DMSO) at a concentration of 10 mmol/L.

### Proliferation assay

Cells were routinely cultured for several passages (up to passage 20) prior to compound testing. On day −1, harvested cells were diluted to 1.25–2.5 × 10^4^ cells/mL, 40 μL added to black 384 well plates (Greiner Bio-One Ltd, Stonehouse, #781090) and incubated overnight at 37 °C, 5% CO_2_. Monotherapy experiments were dosed using an HP D300E dispenser (Tecan UK Ltd, Reading). An ECHO 555 was used to dose combination experiments. At the time of dosing (day 0) and after 120 h for compound-treated plates, 5 µL of SYTOX™ Green Nucleic Acid Stain (Thermo Fisher Scientific S7020) was added and incubated in the dark 1 h at room temperature. Plates were scanned on the Acumen Cellista (SPT Labtech, Melbourn) to quantitate dead cells. Following the addition of 10 µL Saponin, plates were incubated at room temperature for 16 h and re-scanned on the Acumen Cellista to generate a total cell count. Live cell count = Total cell count − Dead cell count. The monotherapy cell growth was calculated as a % of the DMSO control. The combination analysis was carried out in the Genedata Screener compound synergy extension to generate heatmaps and calculate the HSA synergy score.

### Incucyte growth assay

Cells harvested and diluted to 1.0 × 10^4^ cells/mL. Then, 100 μL was added to black 96 well plate (Sigma, Gillingham, Corning Costar #3904) for each cell line and incubated for an hour/overnight at 37 °C, 5% CO_2_. Next, 100 μL growth media was added to give the final plate volume of 200 μL. Compound was added from 10 mM capivasertib and 0.1 mM DMSO fulvestrant using an HP D300E dispenser. Cell confluence reads were taken every 8 h for 96 h at 37 °C, 5% CO_2_ using an Incucyte (Sartorius, UK). After 4 days, the media was replaced and fulvestrant added, capivasertib dosed wells were replaced with DMSO to mimic the 4 days on/3 days off schedule. Dosing was repeated for 3 cycles with reads taken every 8 h. Cell confluence was plotted in GraphPad PRISM 9.0.0. Cell confluence at the end of dosing (or when mean DMSO controls reached 80% confluence) was plotted to carry out statistical analysis between the various treatment groups.

### Western blot analysis of compound-treated cell lines

For this, 1–2 weeks prior to lysate generation as indicated, palbociclib-resistant cells were cultured in T75 flasks, one with 1 or 3 μM palbociclib for MCF7 and T47D cells, respectively, and one flask with palbociclib removed. Parental cells were cultured as normal. Cells were then re-suspended and 8 × 10^5^ cells was added to 6 well plates (Sigma, Gillingham, Corning Costar #3506) and incubated at 37 °C, 5% CO_2_ overnight. Parental cells were treated with 3 μM palbociclib 24 h before lysis. PalboR pools were plated ±1/3 μM Palbociclib. For western analysis of capivasertib, fulvestrant monotherapy and combination treatment, all cells were plated without palbociclib for 96 h prior to drug treatment. All cells were lysed in RIPA lysis buffer (Thermo Fisher Scientific #89901) with 1:100 HALT protease, phosphatase inhibitors (Thermo Fisher Scientific 78442) and 1:5000 Bezonase (Sigma, Gillingham, E1014). Cleared lysates were quantified for protein concentration (BIORAD, Watford, 500–0112) and diluted with distilled water and 4xLDS loading buffer to 1.33 µg/µL and resolved using NuPAGE 4–12% Bis-Tris Midi Gels (Thermo Fisher Scientific WG1402BOX), run at 150 v for 90 mins. Western transfer was carried out using the iBlot 10-min 20 V program. Membranes were cut into strips and probed with primary antibodies (Supplementary Table 13) diluted in TBS + 0.05% polysorbate (TBST) + 5% Marvel overnight, and then HRP-Goat anti-mouse secondary (CST #7076) or HRP-Goat anti-rabbit secondary (CST #7074) for 1 h. Antibody binding was detected using Pierce West Dura reagent and imaged using a Gbox (Syngene, Cambridge, UK). The jpeg images were processed in ImageJ. Band quantification was carried out in GeneTools (Syngene) and plotted in GraphPad PRISM 8.

### Cell cycle analysis

Cells were plated at 1.5 × 10^5^/well in 6 well plates in the absence of palbociclib for 96 h prior to 48 h drug treatment. Cell cycle analysis was performed using a Click-iT™ EdU Cell Proliferation Kit for Imaging (Thermo Fisher Scientific, C10340) and Hoechst 33342 (Thermo Fisher Scientific, Invitrogen H3570) prior to fluorescence-activated cell sorting analysis on a FACSymphony (Becton Dickinson, USA). Cell cycle gating was carried out using FlowJo_v10.8.0, and cell cycle distribution was plotted in GraphPad PRISM 8.

### In vivo studies

All animal work was conducted according to AstraZeneca’s Global Bioethics Policy (https://www.astrazeneca.com/content/dam/az/Sustainability/Bioethics_Policy.pdf), in accordance with the PREPARE guidelines and reported in line with the ARRIVE guidelines.

ST3632, ST3932, ST1799/HI/PBR, ST3164B/PBR and ST941/HI/PBR studies were performed under contract with XenoStart (San Antonio, TX, U.S.A.) at AAALAC-accredited facilities. Animal studies were performed in accordance with protocols approved by the START ‘Institutional Animal Care and Use Committee’ (IACUC) along with AstraZeneca’s ‘Platform for Animal Research Tracking aNd External Relationships’ (PARTNER) group. Female Athymic Nude, Outbred Homozygous (Crl:NU(NCr)-Foxn1nu) mice aged 6–12 weeks were purchased from The Jackson Laboratory. Animals were housed at a density of 4–6 animals per cage in individually vented cages enriched with Corncob bedding, nesting sheets and plastic housing. Animals were identified by ear notch and or Lab Stamp® (Charles River). Animals were acclimatised for a minimum of 24 h before entering studies. The housing room temperature was 72 ± 2 °F with humidity at 45 ± 15% with a 12 h light, 12 h dark cycle. Animals were fed ad libitum with an irradiated rodent diet and drinking water. Drinking water was supplemented with β-oestradiol (8.5 mg/l) from when tumours were implanted. Xenografts were established by subcutaneous (SC) surgical implantation of ~70 mg tumour fragment into the right flanks of 6- to 12-week-old female athymic nude mice under anaesthesia (isoflurane). Tumours were allowed to reach 0.15–0.3 cm^3^ before being randomised. For combination studies, *n* = 6 animals in vehicle control arms and *n* = 5 animals in treatment arms. For capivasertib dose-response studies, *n* = 7 animals per arm.

HBCx-34, HBCx-3, HBCx-19, T272, BCx-015-ROU, HBCx-22, BB6RC160 and T486 studies were performed under contract with Xentech under authorisation by the ‘Direction Départementale de la Protection des Populations, Ministère de l’Agriculture et de l’Alimentation’, France and in accordance with protocols approved by Xentech along with AstraZeneca’s PARTNER group. Female Athymic nude -Foxn1^nu^ mice aged 6 to 11 weeks were purchased from ENVIGO, France. Animals were housed at a density of three animals per cage in individual vented cages enriched with sterilised dust-free bedding cobs. Animals were identified via an RFID chip numbering system (Biolog Id TINY). Animals were acclimatised for a week before entering studies. The housing room temperature was 24 ± 2 °C with humidity at 55 ± 15% with a 14 h light, 10 h dark cycle. Animals were fed ad libitum with an irradiated rodent diet and drinking water. Drinking water was supplemented with β-oestradiol (8.5 mg/l) from when tumours were implanted. Xenografts were established by subcutaneous surgical implantation of ~20 mm^3^ into the interscapular region under anaesthesia (Ketamine/Xylazine). Tumours were allowed to reach 0.075–0.256 cm^3^ (0.1–0.3 cm^3^ for HBCx-22) before being randomly assigned into treatment groups. Xentech studies were run with *n* = 3 animals per arm.

CTG-3302 was licensed from Champions Oncology, and studies were performed internally at AstraZeneca in the United States of America in AAALAC-accredited facilities. Animal studies were performed in accordance with protocols approved by the IACUC, AstraZeneca R&D (Boston) in compliance with the Guide for the Care and Use of Laboratory Animals, 8th Edition (National Research Council, National Academies Press, Washington, D.C., USA). Female NSG aged 5–6 weeks were purchased from The Jackson Laboratory. Animals were housed at a density of five animals per cage in individually vented cages enriched with corncob bedding, nesting material and solid plastic enrichment tubes. Animals were acclimatised for a week before entering studies. Animals were identified by LabStamp. The housing room temperature was 72 ± 2 °F with humidity at 57.5 ± 17.5% with a 12 h light, 12 h dark cycle. Animals were fed ad libitum with an irradiated rodent diet and drinking water. In CTG-3302 studies, a 0.18 mg/60 day estrodiol pellet was implanted into animals’ mammary fat pad the day before tumour engraftment under anaesthesia (Isoflurane). Xenografts were established by subcutaneous (SC) surgical implantation of ~30 mm^3^ tumour fragment into the right flank of 5- to 6-week-old female NSG mice. Tumours were allowed to reach 0.1–0.25 cm^3^ before being randomly assigned to study. CTG-3302 studies were run with *n* = 8 animals per arm.

CTC-174 studies were performed internally at AstraZeneca in the United Kingdom under the authorisation of Home Office License PP3292652. Protocols were reviewed by internal review teams independent of the project to ensure compliance with licence PP3292652. Female NSG mice aged 7–13 weeks were purchased from Charles River Labs UK and housed at a density of five animals per cage in individually vented cages, enriched with sterilised dust-free bedding, cardboard house and wooden chew enrichment. Animals were acclimatised for a week before entering studies. Animals were identified via Ear Notch. The housing room temperature was 21 ± 2 °C with humidity at 55 ± 15° C with a 12 h light, 12 h dark cycle. Animals were fed ad libitum with an irradiated rodent diet and drinking water. CTC-174 xenografts were established by surgical implantation of ~3 mm^3^ tumour fragment into the mammary fat pad 9 under anaesthesia (isoflurane). Tumours were allowed to reach 0.25–0.3cm^3^ before being randomised into treatment groups. CTC174 studies were run with *n* = 7–8 animals per arm.

Animals were randomised into treatment groups according to tumour size criteria outlined above to obtain treatment arms with homogeneous geomean volumes. No animal substitutions of additions were carried out. Conscious animals were euthanised by cervical dislocation with secondary confirmation at the end of the study or for welfare condition or weight loss (15% body weight loss for 3 consecutive days or 20% body weight loss).

Tumours were measured twice weekly by calliper to determine width and length measurements. Across all studies, tumour volume was determined by the following calculation:

Volume = (Maximum measurement (length or width) × Minimum measurement (length or width) × Minimum measurement (length or width) × π) / 6000. Data are presented as treatment group geomeans, with error bars depicting SEM as per AstraZeneca best practices. Relative tumour volume (RTV) was calculated using the formula: RTV for day X = (Tumour volume on day X)/(Tumour volume on day 0). Tumour growth inhibition (TGI) was used as a primary outcome measure and was calculated on the last day that >50% of the control group remained in the study. Anticancer effects of capivasertib and fulvestrant were expressed as TGI, which was calculated as follows: Percentage TGI on day X for treatment group = (((Vehicle RTV day X) − (Treatment group RTV day X))/((Vehicle RTV on day X) – (Vehicle RTV on day 0))) × 100. When TGI > 100%, percentage regression on day X for the treatment group = (RTV on day 0 − RTV on day X) × 100. Studies conducted to assess the anti-tumour activity of capivasertib alone and in combination with fulvestrant and were powered to achieve 80% power, with the exception of the Xentech PDX screen models, which ran as a *n* = 3 screening study to assess broad anti-tumour sensitivity across a larger panel of ER+ breast models. Values for volume, RTV and TGI were calculated in Microsoft Excel and plotted in GraphPad PRISM version 9.4.0. Significant *p*-values for TGI relative to vehicle-treated controls were obtained from a one-tailed two-sample *t*-test with unequal variance, presented in the text and highlighted in the figures.

Capivasertib was formulated once weekly as a solution in 10%DMSO / 25% Kleptose, pH 5 and dosed twice daily (BID) 8 h apart by oral gavage 0.1 mL/10 g of the animal using a schedule of 4 days dosing, 3 days not dosing at either 130 mg/kg, 100 mg/kg or 65 mg/kg (as outlined in individual figures). Fulvestrant was formulated once weekly as a suspension in peanut oil and dosed once weekly (QW) subcutaneously as a fixed dose of 0.1 mL/animal (5 mg/animal). Fulvestrant was dosed 1 h after the morning capivasertib oral dose when in combination. Palbociclib was formulated as a solution in 1% polysorbate 80 (in water) and dosed once daily by oral gavage 0.1 mL/10 g of the animal at either 25 mg/kg or 50 mg/kg (as outlined in individual figures). Control animals were dosed with vehicle equivalent to compound dosing groups. Doses for all compounds are highlighted in individual figures.

PDX models in Fig. 5 and Supplementary Fig. 5 were used as a part of a multi-arm study (with arms of no relevance for this publication) and common control monotherapy arms (e.g., vehicle-treated groups). Hence, the same control group data will be used in different publications.

### Pharmacodynamic assessment

For pharmacodynamic analysis, on the final day of dosing, 4 h after morning oral dosing or at the time points indicated, mice were humanely euthanised, and tumour tissue was collected and immediately snap-frozen in liquid nitrogen before storage at −80 °C. Terminal whole blood was collected by intracardiac puncture into an EDTA-coated microtube, spun at 13,000 RPM for 5 min (4 °C), collecting the plasma, which was immediately frozen at −80 °C. To determine levels of protein of interest in tumour samples, snap-frozen tumour fragments were used, and protein was extracted by adding 900 μL of Extraction buffer (20 mM Tris (pH 7.5) #Sigma T2319, 137 mM NaCl #Sigma S5150, 10% Glycerol #Sigma G5516, 50 mM NaF #Sigma S6776, 1 mM Na3VO4 #Sigma S6508, 1% SDS, 1% NP40 substitute Roche #11754599001) with complete protease inhibitor cocktail (Roche #11836145001; 1 tablet per 50 mL) and phosphatase inhibitor cocktail #3 (Sigma #P0044) with benzonase nuclease (Sigma E1014). Samples were homogenised for 30 s three times at 6.5 m/s in fast-prep machine with an incubation at 4 °C for 5 min between runs. Lysates were then sonicated in a chilled diagenode bioruptor in chilled water bath for five cycles of 30 s on high/30 s off. Lysates were then centrifuged for 10 min at 13000 rpm at 4 °C two times, with a change of tube between runs to discard debris. Lysates were transferred into a new tube, and protein in the supernatant was measured (Thermofisher #23227). Lysates were analysed by western blot as outlined in the western methods.

### Immunohistochemistry

Frozen tumours were sectioned using a cryostat at 10 μm and mounted on glass super frost slides. Antibody staining and detection were performed on the Ventana Discovery Ultra system using the Discovery ChromoMap DAB kit for detection. Frozen slides were thawed at RT and fixed for 10 min in 10% neutral buffered formalin. Following fixation, slides were washed 3 times in 1x PBS for 5 min before loading on the Ventana Discovery Ultra system. For Ki67 Immunochemistry: tissues were blocked for 12 min with Antibody Block (Cat. 760–4204). Ki67 antibody was dispensed on slide at a concentration of 2.0 μg/mL and incubated at RT for 32 min. Primary antibody was detected using Discovery OmniMap anti-Rb HRP (Cat. 760–4311) secondary antibody and incubated for 16 min. Discovery ChromoMap Dab kit was used for detection (Cat. 760–159). Tissues were then counterstained for 12 min with Haematoxylin II (Cat. 790–2208) and 4 min with Blueing Reagent (Cat. 76–2037) before being washed. Anti-Ki67 (30-9) #790-4286, Antibody Block, Discovery OmniMap, anti-Rabbit HRP, Discovery ChromoMap DAB Kit Haematoxylin II and Blueing Reagent were all obtained from Roche Diagnostics, UK. Slides were rinsed in soapy water to remove the liquid coverslip and cover slipped with DPX mountant. Slides were imaged using the Aperio AT2 Microscope slide scanner. Images were uploaded into the HALO image analysis software (Indica Labs v3.5.3577). The tissue regions segmented into tumour and host tissue using a DenseNet (v2) classifier to provide tumour ROI for analysis. The segmentation ROIs were reviewed and corrected manually when appropriate. The tumour ROIs were analysed with the multiplex IHC analysis algorithm (v 3.2.3); all cells were detected, and the IHC-positive cells were identified by thresholding the DAB staining intensity. Results were exported and loaded into Prism for statistical analysis. Statistical methods: ANOVA test, quantified using GraphPad PRISM version 9.4.0 (plotted as mean and standard deviation of the mean), *p < 0.05; **p < 0.01; ***p < 0.005, ****p < 0.001.

### Cell treatment and processing for RNA sequencing

Unless otherwise indicated, RNA sequencing was carried out after 24 h treatment with DMSO, 500 nM capivasertib monotherapy, 1 nM fulvestrant monotherapy and 500 nM capivasertib/1 nM fulvestrant combination therapy in all T47D cell lines. For MCF7 cell lines, 24 h treatment with DMSO, 2 µM capivasertib monotherapy, 10 nM fulvestrant monotherapy and 2 µM capivasertib/10 nM fulvestrant combination therapy was used. Additionally, for PalboR cell pools, RNA was extracted after continuous palbociclib treatment and following Palbociclib removal.

### Preparation and processing for RNA sequencing

A T25 flask was set up for each treatment with 7 × 10^5^ cells/flask, *n* = 3 independent samples for T47D, T47D RB−, T47D CDK6H and MCF7 PacqR. Five samples were generated for MCF7 parental, MCF7 RB− as well as MCF7 PacqR continuous palbociclib only (to account for sequencing failures). Cells were incubated at 37 °C, 5% CO_2_ for 96 h in the absence of palbociclib or with continuous palbociclib treatment in PalboR cells as indicated. Media was removed and relevant compound treatments were added and then incubated at 37 °C, 5% CO^2^ for 24 h. Media was then removed, cells were washed with 3 mL DPBS and 0.5 mL TryplE was added to detach the cells. Cells were re-suspended into growth media total and a cell count was carried out to ensure >1 × 10^6^ total cells. Cells were spun down at 300 × *g* for 5 min, and the cell pellets were placed at −80 °C.

RNA extraction was carried out using the RNeasy kit protocol (Qiagen, Manchester) and the resultant samples were sent for RNA sequencing at Novogene (Cambridge, UK) for the T47D cell lines and Genewiz/Azenta for the MCF7 parental and MCF7 RB− as well as MCF7 PacqR continuous palbociclib only. All MCF7 PacqR samples were sequenced internally at AstraZeneca. Continuous Palbociclib treated samples were run twice in internal and Azenta sequencing platforms so direct comparisons could be made across groups without batch effects. RNA concentration was determined by Qubit Flex Fluorometer (Invitrogen), RNA purity was determined using a NanoDrop Eight (Thermo Scientific) and RNA integrity was measured using a 4200 Tapestation (Agilent). Libraries were prepared using NEBNext Ultra II Directional RNA Library Prep Kit for Illumina (New England BioLabs; #E7760L) or NEBNext® Ultra™ II RNA Library Prep Kit (New England BioLabs; E7770L) as per manufactuer’s guidelines. Ribosomal RNA was removed using the NEBNext® Poly(A) mRNA Magnetic Isolation Module (New England BioLabs; E7490L) or NEBNext® rRNA Depletion Kit (Human/Mouse/Rat) (New England BioLabs; E6310X). 9 samples from MCF7 cell lines did not yield any RNA results and were discarded.

### Quality control

Paired-end RNA sequencing with a read length of 150 bp was performed using Illumina NovaSeq 6000. Library sizes and quantification were determined by 4200 Tapestation (Agilent) and pooled libraries were subsequently pooled as equimolar amounts. Each library was loaded onto one lane of an S4 v1.5 flow cell (300 cycles) (Illumina, #20028312). Quality control and gene expression quantification was completed using an RNAseq pipeline implementing bcbio-nextgen (https://bcbio-nextgen.readthedocs.io/en/latest/). Reads were aligned to HRCh38 homo sapiens genome, with augmentation from Ensembl release 86 using HiSat2. Alignments were evaluated for evenness of coverage, rRNA content, genomic context of alignments and complexity using a combination of FastQC, Qualimap and custom tools. Samples with less than 30X coverage (except in the case of MCF7 RB− where samples were sparse) and high duplication (>30%, except in cases where there were too few samples per group) were removed. Only samples with GC content of between 48 and 55% (inclusive) were kept.

Principal components analysis (PCA) was used to determine batch effects and outliers. Strong batch effects were found in T47D and T47D RB− cell lines as well as T47D CDK6H and MCF7 RB− DMSO-treated only.

After all quality control measures were applied, all T47D and T47D RB− had two samples per treatment group, and T47D CDK6H had three samples per treatment group except in the case of DMSO-treated samples (which had two samples). MCF7 parental cell line had four samples for all treatment groups except for DMSO and fulvestrant monotherapy (which had three samples). MCF7 RB− and MCF7 PacqR had three replicates for all treatment groups except MCF7 RB− capivasertib monotherapy and capivasertib/fulvestrant combination (which had four samples). In total, 10 of 42 of all T47D samples and 6 of 56 of all MCF7 samples were removed.

### Differential expression analysis

Differential expression analysis was completed using DESeq2 (version 1.30.1)^46^ in R (version 4.0.2) and was carried out using DESeq2 default settings. Replicate number was used as a covariate for MCF7 parental cell lines due to a skew found in the PCA. Results were filtered by false discovery rate (FDR) multiple test correction (P_FDR_ < 0.01) and log2 fold change thresholds (−1 > log2FC > 1). Where heatmaps of RNA levels are shown, expression levels were first normalised using variance stabilising transformation (VST) and then converted to a *z*-score.

### Pathway enrichment

Pathway enrichment (or over-representation analysis) was implemented using the Computational Biology for Drug Discovery (CBDD) R package toolkit (version 17.2.1) developed by Clarivate Analytics (www.clarivate.com). Differentially expressed genes were assessed for enrichment in reactome (https://reactome.org/) and hallmark^32^ pathways extracted from the molecular signatures database (msigdb, version 7.5.1)^33^. Differentially expressed genes were separated into positive and negative log2 fold change to observe enrichment as upregulated and downregulated pathways. FDR multiple testing correction was applied (P_FDR_ < 0.05).

### Whole exome sequencing

The whole exome sequencing was carried out using three panels: Aligent SureSelect v7 (T47D parental, T47D RB− and T47D CDK6H), SureSelect v6 (MCF7 parental and MCF7 RB−) and IDT (MCF7 PacqR) and all were aligned to hg38. These were then processed through bcbio v1.2.9 (10.5281/zenodo.3564938).

### Supplementary information

Supplementary Methods Results and Figure Legends

Supplementary Figure 1-7

## Data Availability

RNAseq files are available at ArrayExpress (EMBL) with the accession number E-MTAB-13262. All other datasets generated during the current study are available from the corresponding author upon reasonable request.
