# Author Correction: Combining the AKT inhibitor capivasertib and SERD fulvestrant is effective in palbociclib-resistant ER+ breast cancer preclinical models

**DOI:** 10.1038/s41523-023-00581-8

**Published:** 2023-09-19

**Authors:** Lorna Hopcroft, Eleanor M. Wigmore, Stuart C. Williamson, Susana Ros, Cath Eberlein, Jennifer I. Moss, Jelena Urosevic, Larissa S. Carnevalli, Sara Talbot, Lauren Bradshaw, Catherine Blaker, Sreeharsha Gunda, Venetia Owenson, Scott Hoffmann, Daniel Sutton, Stewart Jones, Richard J. A. Goodwin, Brandon S. Willis, Claire Rooney, Elza C. de Bruin, Simon T. Barry

**Affiliations:** 1 Bioscience Early Oncology, AstraZeneca, Cambridge, UK; 2 Early Data Science, Oncology Data Science, AstraZeneca, Cambridge, UK; 3 Information Technology, AstraZeneca, Chennai, Tamil Nadu India; 4 Imaging Sciences, AstraZeneca, Cambridge, UK; 5 Bioscience Early Oncology, AstraZeneca, Boston, MA USA; 6 Translational Medicine, AstraZeneca, Cambridge, UK

**Keywords:** Breast cancer, Preclinical research

Correction to: *npj Breast Cancer* 10.1038/s41523-023-00571-w, published online 18 August 2023

In this article the author name Elza C de Bruin, was incorrectly written as Elza DeBruin. The original article has been corrected.

