# Supplementary material for: Combining the AKT inhibitor capivasertib and SERD fulvestrant is effective in palbociclib-resistant ER+ breast cancer preclinical models
Source: NPJ Breast Cancer. 2023 Aug 5;9:64. doi: 10.1038/s41523-023-00571-w (PMC10404292; doi:10.1038/s41523-023-00571-w)
Supplement: Supplementary file 1 — Supplementary Methods Results and Figure Legends [file 41523_2023_571_MOESM1_ESM.docx]

**Hopcroft et al Supplementary Information**

**Supplementary Results**

**The capivasertib and fulvestrant combination impacts cell cycle and estrogen response pathway transcriptional changes in palbociclib naïve cells**

To gain greater insight into the impact of capivasertib, fulvestrant and the combination, transcriptomic analysis of the T47D parental was performed. Capivasertib and fulvestrant monotherapy treatment modified 444 & 179 genes respectively with only 48 differentially expressed genes in common (Supp. Fig.3A). Capivasertib downregulated transcriptional programs associated with E2F signalling, cell cycle regulation and DNA repair consistent with the cytostatic effect and impact on cell stress following AKT inhibition (Supp. Fig.3D). Interestingly, over the time course of this experiment while capivasertib upregulated expression of significant numbers of genes these did not associate with specific pathway signatures. Consistent with its mode of action, fulvestrant monotherapy treatment downregulated genes associated with ER signalling (Supp. Fig.3D). Upon combination treatment there was a deeper and more consistent change in relative gene expression of genes modulated by monotherapy capivasertib treatment, and a modest improvement in the modulation of genes controlled by fulvestrant as well as an additional 21 downregulated pathways (Supp. Fig.3E). Combination treatment downregulated expression of a number of genes associated with ER signalling as determined by pathway association analysis. Interestingly, the combination treatment upregulated expression of genes associated with TNFα and NfKB signalling as well as a small number of genes associated with other pathways (Supp. Fig.3D). Collectively these data confirm that, at least in the context of the T47D cell line, targeting AKT and ER mediated signalling are complementary. Targeting both therapeutic nodes in combination increased the degree of regulation (up or down) of expression of genes associated with response to both AKT and ER signalling blockade.

**Supplementary Figure Legends**

**Supplementary Figure 1. Additional characterisation of palbociclib resistant cells**

**(A)** Whole exome sequencing (WES) of parental T47D and MCF7, T47D RB-, T47D CDK6H MCF7 RB-and MCF7 PacqR cells carried out by Cambridge Genomics Services on NextSeq 300 cycle with 2 x 150bp at 100X coverage. Driver mutations (with a tumour allele frequency > 10%) and copy number alterations are shown. **(B)** Additional PI3K pathway and ERα regulated protein biomarkers analysed by western blot in the MCF7 parental treated 24hr with 1µM palbociclib (Treated) vs DMSO (Naive) and palbociclib resistant cells treated continuously with 1µM palbociclib (CP) or following 1 week palbociclib withdrawal (PW), all samples run on the same gel.  **(C and D)** Heatmap shows the mRNA Z-scores (mean expression of condition replicates) for **(Ci)** differentially expressed genes in T47D RB- after continuous palbociclib treatment versus parental DMSO, **(Cii)** differentially expressed genes in T47D CDK6H with continuous palbociclib treatment versus parental DMSO, **(Di)** differentially expressed genes in MCF7 RB- after continuous palbociclib treatment versus parental DMSO and **(D ii)** differentially expressed genes in MCF7 PacqR with continuous palbociclib treatment versus parental DMSO. Each row represents a gene with significant differential expression. The number of genes in common across the comparisons are indicated on the right. Z-scores show relative difference in expression between conditions and the distance from the mean gene expression over each condition, representing relative directional but not absolute expression values.

**Supplementary Figure 2. Statistical analysis of FACS cell cycle phase distribution**

FACS analysis was carried out on parental and palbociclib resistant T47D & MCF7 cells plated in the absence of palbociclib for 96hrs prior to a 48hr dose of capivasertib , fulvestrant and the combination of the 2 agents at the doses indicated. Plotted as mean +/- SD (n= 4 biological replicates). Ordinary one-way ANOVA comparing monotherapy and combination treatments with DMSO control and combination vs monotherapy treatment for % S-phase **(A)**, % GO-G1 phase **(B)**, and **(C)** % G2-M phase. *p<0.05; **p<0.01; ***p<0.005, ****p<0.001. **(D)** T47D DMSO treatment example of FACS cell gating strategy for G0-G1, G2-M and S phase cell populations.

**Supplementary Figure 3. Transcriptomic profile of the capivasertib/fulvestrant combination in the T47D parental cell line. (A)** Significant differentially expressed genes after capivasertib/fulvestrant treatment in parental T47D are shown as z-scores (of the mean expression over condition replicates) with each row representing a single gene with significant differential expression. Columns representing the changes in the same genes are shown for DMSO, capivasertib monotherapy and fulvestrant monotherapy treatment. **(B)** The boxplot shows the difference in log2FC in genes that were uniquely found in capivasertib monotherapy and combination as well as genes that were uniquely found in fulvestrant monotherapy and combination. The plot is split by genes that had a negative log2FC in monotherapy (restricted to log2FC < -1.5, on the left) and genes that had a positive log2FC in the monotherapy (restricted to log2FC > 1.5, on the right). If a downregulated gene has a lower log2FC in the combination than the monotherapy (i.e. a deepening of effect) the log2FC difference value would be lower in value. T-test p-values to compare capivasertib and fulvestrant log2FC differences are shown. A non-significant trend is observed showing a stronger downregulation of genes uniquely downregulated by capivasertib monotherapy in the combination. **(C)** Overlap of downregulated genes enriched in pathways (blue) and upregulated genes enriched in pathways (red) in the comparison of capivasertib monotherapy, fulvestrant monotherapy and capivasertib/fulvestrant combination treatment compared to DMSO in T47D parental cell line. **(D)** Pathway heatmaps representing the top enriched pathways ordered by combined log p-value across the groups are shown. Downregulated pathways in blue (top heatmap) and upregulated pathways in red (bottom heatmap). Comparisons of capivasertib monotherapy, fulvestrant monotherapy and combination vs DMSO (control) are shown in the T47D parental cell line. Shade represents log p-value, the numbers to the right of the heatmap are the total number of genes in the pathway and the numbers in yellow are the DEG found in the pathway. **(E)** The 21 pathways that were uniquely enriched in downregulated genes in capivasertib/fulvestrant combination vs DMSO but not in monotherapy are shown in this bubble plot. The colours represent the q-value (or adjusted p-value) and the size represents the gene ratio (i.e. the number of DEG found in the pathway). The pathways are ordered by q-value with the most significant pathways at the top.

**Supplementary Figure 4. Transcriptomic profile of the capivasertib/fulvestrant combination in the MCF7 parental cell line. (A)** Significant differentially expressed genes after capivasertib/fulvestrant treatment in parental MCF7 are shown as z-scores (of the mean expression over condition replicates) with each row representing a single gene with significant differential expression. Columns representing the changes in the same genes are shown for DMSO, capivasertib monotherapy and fulvestrant monotherapy treatment. **(B)** The boxplot shows the difference in log2FC in genes that were uniquely found in capivasertib monotherapy and combination and genes that were uniquely found in fulvestrant monotherapy and combination. The plot is split by genes that had a negative log2FC in monotherapy (restricted to log2FC < -1.5, on the left) and genes that had a positive log2FC in the monotherapy (restricted to log2FC > 1.5, on the right). If a downregulated gene has a lower log2FC in the combination than the monotherapy (i.e. a deepening of effect) the log2FC difference value would be lower in value. T-test p-values to compare capivasertib and fulvestrant log2FC differences are shown. A significant strengthening of the downregulation of genes uniquely downregulated by fulvestrant monotherapy is found in the combination. **(C)** Overlap of downregulated genes enriched in pathways (blue) and upregulated genes enriched in pathways (red) in the comparison of capivasertib monotherapy, fulvestrant monotherapy and capivasertib/fulvestrant combination treatment compared to DMSO in the MCF7 parental cell line. **(D)** Pathway heatmaps representing the top enriched pathways ordered by combined log p-value across the groups are shown. Downregulated pathways blue (top heatmap) and upregulated pathways red (bottom heatmap). Comparisons of capivasertib monotherapy, fulvestrant monotherapy and combination vs DMSO (control) are shown in the MCF7 parental cell line. Shade represents log p-value, the numbers to the right of the heatmap are the total number of genes in the pathway and the numbers in yellow are the DEG found in the pathway **(E)** The top 25 pathways (by q-value) that were uniquely enriched in downregulated genes in capivasertib/fulvestrant combination vs DMSO but not in monotherapy are shown in this bubble plot. The colours represent the q-value (or adjusted p-value) and the size represents the gene ratio (i.e. the number of DEG found in the pathway). The pathways are ordered by q-value with the most significant pathways at the top.

**Supplementary Figure 5. Transcriptomic profile of the capivasertib and fulvestrant monotherapy in T47D/MCF7 parental versus T47D/MCF7 palbociclib resistant cells. (A-D)** Heatmaps represent differentially expressed genes (DEG) in the parental cell lines and are shown as z-scores (of the mean expression over condition replicates) with each row representing a single gene with significant differential expression. Columns representing the relative expression in the same genes in the resistant cell lines (T47D RB- and T47D CDK6H or MCF7 RB- and MCF7 PacqR) with the same treatment are additionally shown for comparison. The number of common genes that were also found to be significantly differentially expressed in PalboR lines are shown below the relevant columns. The heatmaps demonstrate the relative expression of monotherapies in: **(A)** T47D DEG after capivasertib monotherapy (DEG n=483), with 29 genes out of a total of 72 DEG common in T47D RB- capivasertib monotherapy compared to DMSO and 20 of 66 DEG in T47D CDK6H; **(B)** T47D DEG after fulvestrant monotherapy (DEG n=217), with 39 genes out of a total of 51 DEG common in T47D RB- fulvestrant monotherapy compared to DMSO and 48 of 97 DEG in T47D CDK6H; **(C)** MCF7 DEG after capivasertib monotherapy (DEG n=441), with 74 genes out of a total of 401 DEG common in MCF7 RB- capivasertib monotherapy compared to DMSO and 35 of 369 DEG in MCF7 PacqR and **(D)** MCF7 DEG after fulvestrant monotherapy (DEG n=1161), with 48 genes out of a total of 107 DEG common in MCF7 RB- capivasertib monotherapy compared to DMSO and 255 of 1056 DEG in MCF7 PacqR.

**Supplementary Figure 6. The anti-tumour activity of palbociclib *in vivo.* (A)** *In vivo* sensitivity to palbociclib in PDX models ST3632, ST3932, ST1799/HI/PBR, ST3164/PBR and ST941/HI/PBR. Tumours were treated with vehicle (closed circles) or palbociclib (open diamond). All models received 50mg/kg once daily with the exception of CTC174 received 25mg/kg once daily. Line graphs depict geometric means, error bars represent SEM.

**Supplementary Figure 7. The combination of capivasertib and fulvestrant give anti-tumour efficacy *in vivo.* (A)** Heat map depicting tumour TGI across a panel of PDX in response to palbociclib, fulvestrant, capivasertib, or fulvestrant and capivasertib. Right hand boxes indicate mutational status of models for ESR1, AKT, PIK3CA, PTEN and RB1. Numbers by PDX models name indicate individual schedules used in study, using maximum dose tolerated for mouse strain, (1) Palbociclib 50mg/kg, capivasertib 130mg/kg, fulvestrant 5mg/animal, (2) palbociclib 25mg/kg, capivasertib 100mg/kg, fulvestrant 5mg/animal, (3) palbociclib 25mg/kg, capivasertib 130mg/kg, fulvestrant 5mg/animal. Statistical analysis used was one tailed t-test versus vehicle, * P<0.05, **P<0.01. ***P<0.005 **(B)** Dose response of capivasertib with and without a fixed dose of fulvestrant in ST1799 PBR. Line graphs depict geometric means, error bars represent SEM. Left hand plot capivasertib monotherapy groups, right hand plot shows fulvestrant monotherapy and capivasertib combination groups. **(C)** ST1799PBR PDX PIK3CAm E542K: Pharmacodynamic changes phosphorylation and total protein levels of the AKT and mTOR downstream substrates and regulators AKT (Ser473), PRAS40 (Thr246), S6 (Ser235/236) after 4 days of treatment. Data was normalised to the housekeeping protein β-actin and percentage of control plotted as mean and standard deviation of the mean (n = 5). Statistical analysis used was ANOVA test vs vehicle treated, *p<0.05; **p<0.005; ***p<0.0005. Ki-67 was visualized using immunohistochemistry of tumour samples; results were exported and loaded into Prism for statistical analysis, plotted as mean and standard deviation of the mean (n = 5). Statistical analysis used was ANOVA test vs vehicle *p<0.05; **p<0.01; ***p<0.005, ****p<0.001.

**Supplementary Table 1. Genes within the Hallmark Estrogen Response Early pathway that were significantly expressed in PalboR cell lines.**

| **Cell line** | **Direction** | **DEG/Total** | **DEG found in pathway** |
| --- | --- | --- | --- |
| T47D RB- | Upregulated | 19/200 | OLFML3, MAPT, AREG, MICB, FHL2, GAB2, SLC1A4, TSKU, PRSS23, TFF1, ENDOD1, DEPTOR, KLF4, REEP1, SLC27A2, GJA1, ADCY1, PMAIP1, CYP26B1 |
| T47D CDK6H | Upregulated | 21/200 | OLFML3, CCND1, WFS1, CA12, PTGES, MAPT, DEPTOR, CCN5, HSPB8, ELF3, ENDOD1, PRSS23, DLC1, HR, SLC7A2, REEP1, EEIG1, KLK10, KLF4, SLC1A1, NAV2 |
| MCF7 RB- | Upregulated | 45/200 | SLC7A5, KCNK15, TFF1, SYT12, AREG, TSKU, BHLHE40, DHRS2, SLC27A2, HSPB8, CCN5, SCNN1A, DHRS3, MYC, FASN, HR, CBFA2T3, FHL2, ABCA3, RET, TBC1D30, SEC14L2, TMPRSS3, FARP1, SCARB1, CA12, SLC1A4, CLDN7, SYNGR1, HES1, SLC1A1, LAD1, BAG1, INHBB, B4GALT1, EGR3, P2RY2, TIAM1, TFF3, BCL11B, MSMB, DLC1, FCMR, LRIG1, AR |
| MCF7 PacqR | Upregulated | 40/200 | TFF1, SLC7A5, AREG, BAG1, HSPB8, SLC2A1, KCNK15, TBC1D30, FASN, DHRS2, TFF3, ABCA3, AQP3, KCNK5, TMPRSS3, AR, SLC1A1, MSMB, SYT12, MUC1, SLC27A2, WFS1, SLC26A2, B4GALT1, HR, TSKU, RET, SEC14L2, KRT15, P2RY2, SLC1A4, INHBB, FCMR, LRIG1, REEP1, FHL2, OLFML3, BCL11B, ABLIM1, SYNGR1 |
| MCF7 RB- | Downregulated | 60/200 | GFRA1, SLC39A6, ELOVL2, NPY1R, SLC24A3, CALCR, PAPSS2, ADCY1, ZNF185, PGR, GLA, TMEM164, RHOBTB3, EEIG1, SOX3, MYOF, ELF1, RASGRP1, IGFBP4, KLF4, CXCL12, MUC1, IGF1R, TPBG, KRT13, SLC7A2, MLPH, MYBL1, NRIP1, ABHD2, MPPED2, FKBP4, UGCG, RAB31, GAB2, DEPTOR, BLVRB, ELF3, SYBU, JAK2, DYNLT3, KLK10, SULT2B1, AMFR, RAB17, OLFM1, BCL2, CYP26B1, PPIF, RPS6KA2, FRK, MINDY1, MICB, PLAAT3, FOXC1, ADD3, PEX11A, ARL3, SNX24, SLC26A2 |
| MCF7 PacqR | Downregulated | 53/200 | ADCY1, CALCR, RAB31, GFRA1, NPY1R, RHOBTB3, ZNF185, SCNN1A, RASGRP1, ELF3, KLF4, EEIG1, MLPH, PODXL, SLC39A6, ELOVL2, TMEM164, IGF1R, SLC7A2, UGCG, SOX3, PAPSS2, NRIP1, MYOF, PRSS23, KRT13, MYB, CELSR2, GLA, CXCL12, PPIF, BLVRB, ELF1, OLFM1, DHRS3, IGFBP4, FOXC1, FOS, ALDH3B1, AKAP1, DYNLT3, TGIF2, SEMA3B, PTGES, RAB17, SYBU, MYBL1, DLC1, PMAIP1, CD44, DEPTOR, SULT2B1, CCN5 |

Pathway enrichment in the Hallmark estrogen response early pathway was completed on genes that were significantly downregulated or upregulated in PalboR cell lines after continuous palbociclib compared to parental DMSO in both T47D and MCF7. There were 200 total genes within the pathway and the significant differentially expressed genes only are shown. Only those comparisons with a significant enrichment in the pathway are shown. Abbreviations: DEG, differentially expressed genes.

**Supplementary Table 2. Genes within the Hallmark TNFα signaling via NFKB pathway that were significantly expressed in PalboR cell lines (continuous palbociclib vs parental DMSO).**

| **Cell line** | **Direction** | **DEG/Total** | **DEG found in pathway** |
| --- | --- | --- | --- |
| T47D RB- | Upregulated | 15/200 | PLK2, CDKN1A, AREG, SAT1, BTG2, LAMB3, GADD45A, KLF4, SLC16A6, SPHK1, TNFAIP8, BIRC3, PLAU, TNC, PHLDA2 |
| T47D CDK6H | Upregulated | 26/200 | PLK2, CCND1, PMEPA1, LAMB3, FJX1, VEGFA, NR4A1, SAT1, PLAU, SGK1, PER1, SPHK1, RIGI, GADD45A, GEM, INHBA, ETS2, CSF1, DUSP4, PLPP3, MXD1, ZC3H12A, TNC, CCL2, KLF4, IFIT2 |
| MCF7 RB- | Upregulated | 47/200 | AREG, PMEPA1, DUSP4, PFKFB3, BHLHE40, FJX1, MYC, DRAM1, DUSP5, LOC102724428, VEGFA, SPHK1, NFKB2, IER3, NR4A1, CCN1, IRS2, HES1, KDM6B, LITAF, KLF6, RCAN1, SLC2A6, B4GALT1, EGR3, BTG2, PHLDA2, LIF, GEM, TNFAIP8, IER5, ETS2, NINJ1, F2RL1, EFNA1, BCL6, KLF2, IL15RA, IFIH1, SAT1, MARCKS, SOD2, ICOSLG, PDE4B, HBEGF, FOSL1, MAFF |
| MCF7 PacqR | Upregulated | 32/200 | AREG, DUSP4, IFIH1, TAP1, RIGI, RCAN1, RHOB, NFKB2, FJX1, IER3, IFIT2, SPHK1, B4GALT1, PTGER4, DRAM1, GEM, VEGFA, BCL6, KDM6B, KLF6, ACKR3, PFKFB3, SLC2A6, DUSP5, EDN1, PDE4B, NR4A1, CCL5, MAP2K3, KLF2, SOD2, IL15RA |

Pathway enrichment in the Hallmark TNFα signalling via NFKB pathway was completed on genes that were significantly downregulated or upregulated in PalboR cell lines after continuous palbociclib compared to parental DMSO in both T47D and MCF7. There were 200 total genes within the pathway and the significant differentially expressed genes only are shown. Only those comparisons with a significant enrichment in the pathway are shown. Abbreviations: DEG, differentially expressed genes.

**Supplementary Table 3. Pathways overlapping between T47D parental, T47D RB- and T47D CDK6H that were enriched in downregulated genes in comparison of capivasertib/fulvestrant combination treatment versus DMSO.**

|  |  | **T47D parental** | | **T47D RB-** | | **T47D CDK6H** | |
| --- | --- | --- | --- | --- | --- | --- | --- |
| **Ontology Term** | **Total genes in pathway** | **DEG Found in Pathway** | **qvalue** | **DEG Found in Pathway** | **qvalue** | **DEG Found in Pathway** | **qvalue** |
| HALLMARK_ESTROGEN_RESPONSE_EARLY | 200 | 49 | 1.43E-22 | 18 | 2.12e-9 | 19 | 5.23e-11 |
| HALLMARK_ESTROGEN_RESPONSE_LATE | 200 | 54 | 4.45E-27 | 23 | 1.16e-14 | 20 | 7.92e-12 |
| HALLMARK_MTORC1_SIGNALING | 200 | 41 | 6.01E-16 | 4 | 0.0444 | 7 | 0.000014 |
| REACTOME_ACTIVATION_OF_ATR_IN_RESPONSE_TO_REPLICATION_STRESS | 37 | 22 | 1.37E-19 | 8 | 0.0292 | 8 | 0.0147 |
| REACTOME_ACTIVATION_OF_THE_PRE_REPLICATIVE_COMPLEX | 33 | 22 | 4.02E-21 | 5 | 0.0093 | 5 | 0.00523 |
| REACTOME_MITOTIC_G1_PHASE_AND_G1_S_TRANSITION | 149 | 41 | 7.32E-21 | 13 | 0.0000926 | 13 | 0.0000311 |

Pathways that were significantly enriched in downregulated genes in comparison of capivasertib/fulvestrant combination therapy vs DMSO (control) in T47D parental, T47D RB- and T47D CDK6H are shown. The pathway name and total number of genes in the pathway are shown on the left. The columns after this refer to the specific cell line and show the numbers of differentially expressed genes from the comparison that are found in the pathway along with the q-value (adjusted p-value). Abbreviations: DEG, differentially expressed genes.

**Supplementary Table 4. Pathways overlapping between T47D parental and T47D RB- but not T47D CDK6H that were enriched in downregulated genes in comparison of capivasertib/fulvestrant combination treatment versus DMSO.**

|  |  | **T47D Parental** | | **T47D RB-** | |
| --- | --- | --- | --- | --- | --- |
| **Ontology Term** | **Total genes in pathway** | **DEG found in pathway** | **qvalue** | **DEG found in pathway** | **qvalue** |
| REACTOME_G2_M_CHECKPOINTS | 166 | 36 | 8.37E-15 | 8 | 0.0444 |
| REACTOME_G1_S_SPECIFIC_TRANSCRIPTION | 29 | 15 | 3.42E-12 | 6 | 0.000192 |
| HALLMARK_MYC_TARGETS_V1 | 200 | 31 | 7.16E-09 | 11 | 0.00263 |
| REACTOME_G0_AND_EARLY_G1 | 27 | 12 | 8.10E-09 | 4 | 0.0292 |
| HALLMARK_MYC_TARGETS_V2 | 58 | 16 | 3.38E-08 | 6 | 0.0085 |
| REACTOME_CDC6_ASSOCIATION_WITH_THE_ORC_ORIGIN_COMPLEX | 11 | 6 | 4.39709E-05 | 3 | 0.0292 |
| HALLMARK_UV_RESPONSE_UP | 158 | 19 | 0.000552494 | 8 | 0.0352 |

Pathways that were significantly enriched in downregulated genes in comparison of capivasertib/fulvestrant combination therapy vs DMSO (control) in T47D parental and T47D RB- (but not T47D CDK6H) are shown. The pathway name and total number of genes in the pathway are shown on the left. The columns after this refer to the specific cell line and show the numbers of differentially expressed genes from the comparison that are found in the pathway along with the q-value (adjusted p-value). Abbreviations: DEG, differentially expressed genes.

**Supplementary Table 5. Pathways overlapping between T47D parental and T47D CDK6H but not T47D RB- that were enriched in downregulated genes in comparison of capivasertib/fulvestrant combination treatment versus DMSO.**

|  |  | **T47D Parental** | | **T47D CDK6H** | |
| --- | --- | --- | --- | --- | --- |
| **Ontology Term** | **Total genes in pathway** | **DEG found in pathway** | **qvalue** | **DEG found in pathway** | **qvalue** |
| HALLMARK_G2M_CHECKPOINT | 200 | 86 | 1.17E-63 | 19 | 5.23e-11 |
| REACTOME_DNA_REPLICATION | 128 | 43 | 1.29E-25 | 20 | 7.92e-12 |
| REACTOME_DNA_REPLICATION_PRE_INITIATION | 85 | 24 | 1.74E-12 | 7 | 0.000014 |
| REACTOME_CHOLESTEROL_BIOSYNTHESIS | 25 | 8 | 0.00010135 | 8 | 0.0147 |
| HALLMARK_CHOLESTEROL_HOMEOSTASIS | 74 | 12 | 0.000866471 | 5 | 0.00523 |
| REACTOME_METABOLISM_OF_STEROIDS | 151 | 14 | 0.046894979 | 13 | 0.0000311 |

Pathways that were significantly enriched in downregulated genes in comparison of capivasertib/fulvestrant combination therapy vs DMSO (control) in T47D parental and T47D CDK6H (but not T47D RB-) are shown. The pathway name and total number of genes in the pathway are shown on the left. The columns after this refer to the specific cell line and show the numbers of differentially expressed genes from the comparison that are found in the pathway along with the q-value (adjusted p-value). Abbreviations: DEG, differentially expressed genes.

**Supplementary Table 6. Pathways overlapping between T47D Parental and T47D RB- but not T47D CDK6H that were enriched in upregulated genes in comparison of capivasertib/fulvestrant combination treatment versus DMSO.**

|  |  | **T47D Parental** | | **T47D RB-** | | |
| --- | --- | --- | --- | --- | --- | --- |
| **Ontology Term** | **Total genes in pathway** | **DEG found in pathway** | **qvalue** | **DEG found in pathway** | | **qvalue** |
| HALLMARK_HYPOXIA | 200 | 15 | 0.0452 | 9 | 0.040 | |
|  |  |  |  |  |  |  |

Pathways that were significantly enriched in upregulated genes in comparison of capivasertib/fulvestrant combination therapy vs DMSO (control) in T47D parental and T47D RB- (but not T47D CDK6H) are shown. The pathway name and total number of genes in the pathway are shown on the left. The columns after this refer to the specific cell line and show the numbers of differentially expressed genes from the comparison that are found in the pathway along with the q-value (adjusted p-value). Abbreviations: DEG, differentially expressed genes.

**Supplementary Table 7. Pathways overlapping between MCF7 parental, MCF7 RB- and MCF7 PacqR that were enriched in downregulated genes in comparison of capivasertib/fulvestrant combination treatment versus DMSO.**

|  |  | **MCF7 parental** | | **MCF7 RB-** | | **MCF7 PacqR** | |
| --- | --- | --- | --- | --- | --- | --- | --- |
| **Ontology Term** | **Total genes in pathway** | **DEG found in pathway** | **qvalue** | **DEG found in pathway** | **qvalue** | **DEG found in pathway** | **qvalue** |
| REACTOME_CELL_CYCLE | 686 | 214 | 2.47e-93 | 91 | 1.02e-13 | 96 | 5.81e-15 |
| REACTOME_CELL_CYCLE_MITOTIC | 554 | 186 | 3.85e-86 | 76 | 4e-12 | 79 | 1.18e-12 |
| HALLMARK_E2F_TARGETS | 200 | 105 | 5.89e-70 | 58 | 1.58e-24 | 67 | 5.03e-32 |
| REACTOME_CELL_CYCLE_CHECKPOINTS | 290 | 107 | 5.24e-52 | 40 | 0.0000026 | 55 | 1.46e-13 |
| HALLMARK_ESTROGEN_RESPONSE_LATE | 200 | 89 | 4.77e-51 | 44 | 1.13e-13 | 79 | 3.04e-44 |
| REACTOME_M_PHASE | 410 | 120 | 1.93e-46 | 43 | 0.000625 | 43 | 0.00198 |
| HALLMARK_G2M_CHECKPOINT | 200 | 81 | 1.56e-42 | 40 | 4.58e-11 | 57 | 5.76e-23 |
| REACTOME_G2_M_CHECKPOINTS | 166 | 65 | 9.75e-33 | 28 | 0.00000429 | 27 | 0.000028 |
| HALLMARK_ESTROGEN_RESPONSE_EARLY | 200 | 71 | 1.1e-32 | 48 | 1.93e-16 | 90 | 1.51e-56 |
| REACTOME_CHROMOSOME_MAINTENANCE | 136 | 59 | 1.18e-32 | 21 | 0.000389 | 25 | 0.00000795 |
| REACTOME_HOMOLOGY_DIRECTED_REPAIR | 135 | 58 | 7.81e-32 | 27 | 2.03e-7 | 21 | 0.000763 |
| REACTOME_MITOTIC_PROMETAPHASE | 202 | 70 | 1.51e-31 | 24 | 0.00524 | 32 | 0.00000607 |
| REACTOME_DNA_DOUBLE_STRAND_BREAK_REPAIR | 164 | 61 | 2.42e-29 | 30 | 2.33e-7 | 22 | 0.00404 |
| REACTOME_DNA_REPLICATION | 128 | 51 | 4.2e-26 | 29 | 3.13e-9 | 27 | 1.7e-7 |
| REACTOME_DNA_STRAND_ELONGATION | 32 | 27 | 5.11e-26 | 17 | 1.04e-11 | 14 | 5.89e-8 |
| REACTOME_MITOTIC_G1_PHASE_AND_G1_S_TRANSITION | 149 | 54 | 2.17e-25 | 25 | 0.0000187 | 29 | 2.89e-7 |
| REACTOME_S_PHASE | 162 | 54 | 2.37e-23 | 30 | 1.91e-7 | 31 | 1.43e-7 |
| HALLMARK_MTORC1_SIGNALING | 200 | 59 | 1.63e-22 | 72 | 7.74e-38 | 56 | 3.32e-22 |
| REACTOME_G2_M_DNA_DAMAGE_CHECKPOINT | 93 | 40 | 5.18e-22 | 14 | 0.00906 | 13 | 0.0421 |
| REACTOME_PROCESSING_OF_DNA_DOUBLE_STRAND_BREAK_ENDS | 96 | 40 | 2.07e-21 | 15 | 0.00437 | 14 | 0.0216 |
| REACTOME_TELOMERE_MAINTENANCE | 109 | 42 | 6.03e-21 | 19 | 0.000192 | 16 | 0.00966 |
| REACTOME_ACTIVATION_OF_THE_PRE_REPLICATIVE_COMPLEX | 33 | 22 | 1.75e-17 | 12 | 0.00000437 | 13 | 8.66e-7 |
| REACTOME_ACTIVATION_OF_ATR_IN_RESPONSE_TO_REPLICATION_STRESS | 37 | 23 | 2.78e-17 | 15 | 2.64e-8 | 16 | 4.81e-9 |
| REACTOME_DNA_REPLICATION_PRE_INITIATION | 85 | 31 | 9.48e-15 | 16 | 0.000348 | 16 | 0.000687 |
| REACTOME_HOMOLOGOUS_DNA_PAIRING_AND_STRAND_EXCHANGE | 42 | 22 | 1.92e-14 | 12 | 0.0000622 | 14 | 0.0000026 |
| REACTOME_HDR_THROUGH_HOMOLOGOUS_RECOMBINATION_HRR | 66 | 27 | 2.56e-14 | 16 | 0.0000165 | 16 | 0.0000278 |
| REACTOME_UNWINDING_OF_DNA | 12 | 12 | 1.29e-13 | 10 | 1.56e-9 | 7 | 0.0000501 |
| REACTOME_G1_S_SPECIFIC_TRANSCRIPTION | 29 | 18 | 1.38e-13 | 10 | 0.0000666 | 10 | 0.000102 |
| REACTOME_LAGGING_STRAND_SYNTHESIS | 20 | 15 | 3.38e-13 | 7 | 0.00159 | 7 | 0.00241 |
| REACTOME_TELOMERE_C_STRAND_LAGGING_STRAND_SYNTHESIS | 34 | 17 | 7.63e-11 | 9 | 0.00164 | 9 | 0.00263 |
| REACTOME_RESOLUTION_OF_D_LOOP_STRUCTURES_THROUGH_SYNTHESIS_DEPENDENT_STRAND_ANNEALING_SDSA | 26 | 15 | 9.16e-11 | 9 | 0.000192 | 10 | 0.0000354 |
| REACTOME_G0_AND_EARLY_G1 | 27 | 15 | 1.88e-10 | 7 | 0.00925 | 10 | 0.0000501 |
| REACTOME_EXTENSION_OF_TELOMERES | 51 | 20 | 2.4e-10 | 13 | 0.0000868 | 14 | 0.0000278 |
| REACTOME_POLYMERASE_SWITCHING | 14 | 11 | 4.05e-10 | 6 | 0.0015 | 6 | 0.00221 |
| REACTOME_RESOLUTION_OF_D_LOOP_STRUCTURES | 33 | 16 | 5.34e-10 | 10 | 0.000213 | 11 | 0.0000501 |
| REACTOME_HDR_THROUGH_SINGLE_STRAND_ANNEALING_SSA | 37 | 16 | 4.33e-9 | 7 | 0.0452 | 10 | 0.000983 |
| HALLMARK_MYC_TARGETS_V1 | 200 | 38 | 1.68e-8 | 44 | 1.13e-13 | 32 | 0.00000498 |
| REACTOME_ASSEMBLY_OF_THE_PRE_REPLICATIVE_COMPLEX | 68 | 20 | 7.14e-8 | 13 | 0.00164 | 12 | 0.00966 |
| REACTOME_SWITCHING_OF_ORIGINS_TO_A_POST_REPLICATIVE_STATE | 91 | 23 | 1.28e-7 | 13 | 0.0206 | 15 | 0.00465 |
| REACTOME_POLYMERASE_SWITCHING_ON_THE_C_STRAND_OF_THE_TELOMERE | 26 | 12 | 2.72e-7 | 7 | 0.00757 | 7 | 0.0113 |
| REACTOME_E2F_MEDIATED_REGULATION_OF_DNA_REPLICATION | 22 | 11 | 3.71e-7 | 6 | 0.0166 | 8 | 0.000623 |
| REACTOME_ORC1_REMOVAL_FROM_CHROMATIN | 71 | 19 | 8.61e-7 | 11 | 0.0237 | 12 | 0.0133 |
| REACTOME_CHOLESTEROL_BIOSYNTHESIS | 25 | 11 | 0.00000183 | 11 | 0.00000176 | 7 | 0.00949 |
| REACTOME_TRANSCRIPTION_OF_E2F_TARGETS_UNDER_NEGATIVE_CONTROL_BY_DREAM_COMPLEX | 19 | 9 | 0.0000113 | 5 | 0.0411 | 6 | 0.0113 |
| REACTOME_CDC6_ASSOCIATION_WITH_THE_ORC_ORIGIN_COMPLEX | 11 | 7 | 0.0000141 | 6 | 0.000312 | 6 | 0.000486 |
| HALLMARK_UV_RESPONSE_UP | 158 | 27 | 0.0000288 | 23 | 0.000402 | 21 | 0.00593 |
| HALLMARK_GLYCOLYSIS | 200 | 30 | 0.00011 | 30 | 0.0000169 | 25 | 0.00438 |
| HALLMARK_MYC_TARGETS_V2 | 58 | 14 | 0.000128 | 30 | 1.62e-20 | 25 | 2.48e-14 |
| HALLMARK_CHOLESTEROL_HOMEOSTASIS | 74 | 15 | 0.000529 | 17 | 0.0000165 | 15 | 0.00052 |
| HALLMARK_HYPOXIA | 200 | 27 | 0.00154 | 28 | 0.000124 | 24 | 0.00966 |

Pathways that were significantly enriched in downregulated genes in comparison of capivasertib/fulvestrant combination therapy vs DMSO (control) in MCF7 parental, MCF7 RB- and MCF7 PacqR are shown. The pathway name and total number of genes in the pathway are shown on the left. The columns after this refer to the specific cell line and show the numbers of differentially expressed genes from the comparison that are found in the pathway along with the q-value (adjusted p-value). Abbreviations: DEG, differentially expressed genes.

**Supplementary Table 8. Pathways overlapping between MCF7 parental and MCF7 RB- but not MCF7 PacqR that were enriched in downregulated genes in comparison of capivasertib/fulvestrant combination treatment versus DMSO.**

|  |  | **MCF7 parental** | | **MCF7 RB-** | |
| --- | --- | --- | --- | --- | --- |
| **Ontology Term** | **Total genes in pathway** | **DEG found in pathway** | **qvalue** | **DEGfound in pathway** | **qvalue** |
| REACTOME_DNA_REPAIR | 328 | 82 | 5.11E-26 | 40 | 0.0000513 |
| REACTOME_HCMV_EARLY_EVENTS | 130 | 48 | 6.28E-23 | 17 | 0.012 |
| REACTOME_MEIOTIC_RECOMBINATION | 81 | 38 | 1.60E-22 | 14 | 0.00255 |
| REACTOME_BASE_EXCISION_REPAIR | 88 | 38 | 5.29E-21 | 15 | 0.00179 |
| REACTOME_CONDENSATION_OF_PROPHASE_CHROMOSOMES | 68 | 33 | 3.42E-20 | 12 | 0.00576 |
| REACTOME_MEIOSIS | 113 | 41 | 2.57E-19 | 15 | 0.0197 |
| REACTOME_MITOTIC_PROPHASE | 137 | 45 | 3.10E-19 | 19 | 0.00349 |
| REACTOME_DISEASES_OF_PROGRAMMED_CELL_DEATH | 96 | 36 | 1.70E-17 | 15 | 0.00437 |
| REACTOME_PRC2_METHYLATES_HISTONES_AND_DNA | 67 | 30 | 3.75E-17 | 10 | 0.042 |
| REACTOME_DNA_METHYLATION | 59 | 28 | 7.70E-17 | 11 | 0.00622 |
| REACTOME_TRANSCRIPTIONAL_REGULATION_OF_GRANULOPOIESIS | 84 | 33 | 8.68E-17 | 15 | 0.00112 |
| REACTOME_SIRT1_NEGATIVELY_REGULATES_RRNA_EXPRESSION | 62 | 28 | 3.86E-16 | 10 | 0.0269 |
| REACTOME_TRANSCRIPTIONAL_REGULATION_BY_SMALL_RNAS | 101 | 35 | 8.27E-16 | 15 | 0.007 |
| REACTOME_REPRODUCTION | 139 | 41 | 1.08E-15 | 16 | 0.0456 |
| REACTOME_HCMV_LATE_EVENTS | 109 | 36 | 1.60E-15 | 14 | 0.032 |
| REACTOME_ACTIVATED_PKN1_STIMULATES_TRANSCRIPTION_OF_AR_ANDROGEN_RECEPTOR_REGULATED_GENES_KLK2_AND_KLK3 | 61 | 27 | 2.47E-15 | 10 | 0.0241 |
| REACTOME_ESTROGEN_DEPENDENT_GENE_EXPRESSION | 144 | 41 | 4.02E-15 | 21 | 0.000876 |
| REACTOME_ERCC6_CSB_AND_EHMT2_G9A_POSITIVELY_REGULATE_RRNA_EXPRESSION | 70 | 28 | 1.56E-14 | 11 | 0.0219 |
| REACTOME_GENE_SILENCING_BY_RNA | 135 | 37 | 3.91E-13 | 17 | 0.0174 |
| REACTOME_B_WICH_COMPLEX_POSITIVELY_REGULATES_RRNA_EXPRESSION | 85 | 29 | 5.39E-13 | 14 | 0.00415 |
| REACTOME_RNA_POLYMERASE_I_PROMOTER_ESCAPE | 85 | 28 | 3.87E-12 | 12 | 0.0298 |
| REACTOME_POSITIVE_EPIGENETIC_REGULATION_OF_RRNA_EXPRESSION | 100 | 30 | 8.30E-12 | 15 | 0.00635 |
| REACTOME_ESR_MEDIATED_SIGNALING | 215 | 45 | 2.01E-11 | 26 | 0.00245 |
| REACTOME_FORMATION_OF_THE_BETA_CATENIN_TCF_TRANSACTIVATING_COMPLEX | 86 | 27 | 3.58E-11 | 12 | 0.032 |
| REACTOME_RMTS_METHYLATE_HISTONE_ARGININES | 73 | 24 | 1.88E-10 | 12 | 0.00994 |
| REACTOME_SIGNALING_BY_NUCLEAR_RECEPTORS | 291 | 51 | 5.82E-10 | 31 | 0.00521 |
| REACTOME_NONHOMOLOGOUS_END_JOINING_NHEJ | 67 | 22 | 1.29E-09 | 10 | 0.042 |
| REACTOME_PROCESSIVE_SYNTHESIS_ON_THE_LAGGING_STRAND | 15 | 11 | 1.35E-09 | 5 | 0.0166 |
| REACTOME_RESOLUTION_OF_ABASIC_SITES_AP_SITES | 38 | 16 | 6.82E-09 | 9 | 0.00374 |
| REACTOME_RESOLUTION_OF_AP_SITES_VIA_THE_MULTIPLE_NUCLEOTIDE_PATCH_REPLACEMENT_PATHWAY | 25 | 13 | 1.16E-08 | 6 | 0.028 |
| REACTOME_FORMATION_OF_TUBULIN_FOLDING_INTERMEDIATES_BY_CCT_TRIC | 25 | 12 | 1.60E-07 | 6 | 0.028 |
| REACTOME_PCNA_DEPENDENT_LONG_PATCH_BASE_EXCISION_REPAIR | 21 | 11 | 2.02E-07 | 6 | 0.013 |
| REACTOME_GAP_FILLING_DNA_REPAIR_SYNTHESIS_AND_LIGATION_IN_GG_NER | 25 | 11 | 1.83E-06 | 6 | 0.028 |
| REACTOME_PROCESSIVE_SYNTHESIS_ON_THE_C_STRAND_OF_THE_TELOMERE | 19 | 8 | 0.000114 | 5 | 0.0411 |
| REACTOME_GLUCOSE_METABOLISM | 92 | 18 | 0.000176 | 20 | 0.00000437 |
| REACTOME_GLYCOLYSIS | 72 | 15 | 0.000391 | 16 | 0.0000514 |
| REACTOME_METABOLISM_OF_NUCLEOTIDES | 98 | 18 | 0.000411 | 20 | 0.0000126 |
| REACTOME_INTERCONVERSION_OF_NUCLEOTIDE_DI_AND_TRIPHOSPHATES | 29 | 9 | 0.000508 | 7 | 0.0139 |
| REACTOME_GLUCONEOGENESIS | 34 | 9 | 0.00177 | 10 | 0.000281 |
| REACTOME_SNRNP_ASSEMBLY | 53 | 11 | 0.00358 | 9 | 0.0298 |
| REACTOME_REGULATION_OF_HSF1_MEDIATED_HEAT_SHOCK_RESPONSE | 82 | 14 | 0.00517 | 12 | 0.0239 |
| REACTOME_ACTIVATION_OF_GENE_EXPRESSION_BY_SREBF_SREBP | 42 | 9 | 0.00862 | 12 | 0.0000622 |
| REACTOME_TRNA_PROCESSING_IN_THE_NUCLEUS | 59 | 11 | 0.00862 | 10 | 0.0204 |
| REACTOME_METABOLISM_OF_CARBOHYDRATES | 293 | 32 | 0.0153 | 36 | 0.00012 |
| REACTOME_TRNA_PROCESSING | 110 | 15 | 0.0302 | 20 | 0.0000638 |
| REACTOME_SCAVENGING_BY_CLASS_F_RECEPTORS | 6 | 3 | 0.0317 | 3 | 0.0411 |

Pathways that were significantly enriched in downregulated genes in comparison of capivasertib/fulvestrant combination therapy vs DMSO (control) in MCF7 parental and MCF7 RB- (but not MCF7 PacqR) are shown. The pathway name and total number of genes in the pathway are shown on the left. The columns after this refer to the specific cell line and show the numbers of differentially expressed genes from the comparison that are found in the pathway along with the q-value (adjusted p-value). Abbreviations: DEG, differentially expressed genes.

**Supplementary Table 9. Pathways overlapping between MCF7 parental and MCF7 PacqR but not MCF7 RB- that were enriched in downregulated genes in comparison of capivasertib/fulvestrant combination treatment versus DMSO.**

|  |  | **MCF7 Parental** | | **MCF7 PacqR** | |
| --- | --- | --- | --- | --- | --- |
| **Ontology Term** | **Total genes in pathway** | **DEG found in pathway** | **qvalue** | **DEG found in pathway** | **qvalue** |
| REACTOME_RESOLUTION_OF_SISTER_CHROMATID_COHESION | 125 | 51 | 1.23E-26 | 27 | 1.1e-7 |
| REACTOME_MITOTIC_METAPHASE_AND_ANAPHASE | 235 | 69 | 3.84E-26 | 35 | 0.00000704 |
| REACTOME_RHO_GTPASE_EFFECTORS | 317 | 80 | 1.13E-25 | 31 | 0.0389 |
| REACTOME_DEPOSITION_OF_NEW_CENPA_CONTAINING_NUCLEOSOMES_AT_THE_CENTROMERE | 70 | 38 | 1.79E-25 | 12 | 0.0118 |
| REACTOME_SEPARATION_OF_SISTER_CHROMATIDS | 190 | 57 | 3.79E-22 | 30 | 0.0000142 |
| REACTOME_RHO_GTPASES_ACTIVATE_FORMINS | 139 | 48 | 1.56E-21 | 25 | 0.0000119 |
| REACTOME_MITOTIC_SPINDLE_CHECKPOINT | 111 | 39 | 8.45E-18 | 26 | 3.99e-8 |
| REACTOME_REGULATION_OF_TP53_ACTIVITY_THROUGH_PHOSPHORYLATION | 92 | 22 | 7.38E-07 | 15 | 0.00518 |
| REACTOME_POLO_LIKE_KINASE_MEDIATED_EVENTS | 16 | 9 | 1.83E-06 | 5 | 0.033 |
| REACTOME_TRANSCRIPTIONAL_REGULATION_BY_TP53 | 363 | 50 | 2.66E-06 | 34 | 0.0475 |
| REACTOME_CONDENSATION_OF_PROMETAPHASE_CHROMOSOMES | 11 | 7 | 1.41E-05 | 4 | 0.0475 |
| REACTOME_TP53_REGULATES_TRANSCRIPTION_OF_CELL_CYCLE_GENES | 49 | 14 | 1.75E-05 | 9 | 0.033 |
| REACTOME_TRANSCRIPTION_OF_E2F_TARGETS_UNDER_NEGATIVE_CONTROL_BY_P107_RBL1_AND_P130_RBL2_IN_COMPLEX_WITH_HDAC1 | 16 | 8 | 2.69E-05 | 5 | 0.033 |
| REACTOME_APC_C_MEDIATED_DEGRADATION_OF_CELL_CYCLE_PROTEINS | 88 | 19 | 2.69E-05 | 14 | 0.00966 |
| REACTOME_TRANSCRIPTIONAL_REGULATION_BY_E2F6 | 34 | 11 | 5.58E-05 | 7 | 0.048 |
| REACTOME_PHOSPHORYLATION_OF_EMI1 | 6 | 5 | 6.60E-05 | 4 | 0.00432 |
| REACTOME_CYCLIN_A_B1_B2_ASSOCIATED_EVENTS_DURING_G2_M_TRANSITION | 25 | 9 | 0.000141 | 7 | 0.00949 |
| REACTOME_G2_M_DNA_REPLICATION_CHECKPOINT | 5 | 4 | 0.000711 | 3 | 0.0359 |
| REACTOME_G2_PHASE | 5 | 4 | 0.000711 | 3 | 0.0359 |
| REACTOME_E2F_ENABLED_INHIBITION_OF_PRE_REPLICATION_COMPLEX_FORMATION | 9 | 5 | 0.000932 | 5 | 0.00207 |
| REACTOME_PHOSPHORYLATION_OF_THE_APC_C | 20 | 5 | 0.0492 | 6 | 0.0147 |

Pathways that were significantly enriched in downregulated genes in comparison of capivasertib/fulvestrant combination therapy vs DMSO (control) in MCF7 parental and MCF7 PacqR (but not MCF7 RB-) are shown. The pathway name and total number of genes in the pathway are shown on the left. The columns after this refer to the specific cell line and show the numbers of differentially expressed genes from the comparison that are found in the pathway along with the q-value (adjusted p-value). Abbreviations: DEG, differentially expressed genes.

**Supplementary Table 10. Pathways overlapping between MCF7 RB- and MCF7 PacqR but not MCF7 Parental that were enriched in downregulated genes in comparison of capivasertib/fulvestrant combination treatment versus DMSO.**

|  |  | **MCF7 RB-** | | **MCF7 PacqR** | |
| --- | --- | --- | --- | --- | --- |
| **Ontology Term** | **Total genes in pathway** | **DEG found in pathway** | **qvalue** | **DEG found in pathway** | **qvalue** |
| HALLMARK_UNFOLDED_PROTEIN_RESPONSE | 113 | 30 | 2.16E-11 | 23 | 0.00000428 |
| REACTOME_CYTOSOLIC_TRNA_AMINOACYLATION | 24 | 6 | 0.0239 | 16 | 7.28e-13 |
| REACTOME_TRNA_AMINOACYLATION | 42 | 8 | 0.0263 | 17 | 4.59e-9 |
| REACTOME_BASIGIN_INTERACTIONS | 25 | 6 | 0.02802 | 6 | 0.045 |

Pathways that were significantly enriched in downregulated genes in comparison of capivasertib/fulvestrant combination therapy vs DMSO (control) in MCF7 RB- and MCF7 PacqR (but not MCF7 Parental) are shown. The pathway name and total number of genes in the pathway are shown on the left. The columns after this refer to the specific cell line and show the numbers of differentially expressed genes from the comparison that are found in the pathway along with the q-value (adjusted p-value). Abbreviations: DEG, differentially expressed genes.

**Supplementary Table 11. Pathways overlapping between MCF7 Parental and MCF7 PacqR but not MCF7 RB- that were enriched in upregulated genes in comparison of capivasertib fulvestrant combination treatment versus DMSO.**

|  |  | **MCF7 RB-** | | **MCF7 PacqR** | |
| --- | --- | --- | --- | --- | --- |
| **Ontology Term** | **Total genes in pathway** | **DEG found in pathway** | **qvalue** | **Total genes in pathway** | **qvalue** |
| HALLMARK_MYOGENESIS | 200 | 22 | 0.0148 | 21 | 0.0465 |

Pathways that were significantly enriched in upregulated genes in comparison of capivasertib/fulvestrant combination therapy vs DMSO (control) in MCF7 parental and MCF7 PacqR (but not MCF7 RB-) are shown. The pathway name and total number of genes in the pathway are shown on the left. The columns after this refer to the specific cell line and show the numbers of differentially expressed genes from the comparison that are found in the pathway along with the q-value (adjusted p-value). Abbreviations: DEG, differentially expressed genes.

**Supplementary Table 12.** **Clinical and genomic characteristics of ER+ breast cancer patient derived xenografts treated with capivasertib and fulvestrant.**

| **ER+ BC PDX model** | **Biopsied** | **PI3KCA/AKT/PTEN** | **ESR1** | **other** |  |
| --- | --- | --- | --- | --- | --- |
| **ST3632​** | Prim​ | AKT1_E17K​ | wt​ | FGFR3amp, ​NF1amp, TP53m |  |
| **ST3932​** | Prim​ | PI3KCA_R88Q​ / PTENdel | wt​ | FGFR2amp, NF1m, Rb1m (M695I), TP53m​ |  |
| **ST1799/HI/PBR​** | Met​ | PI3KCA_E542K​ | wt​ |  |  |
| **CTC174** | CTC | PIK3CA N345K | D538G |  |  |
| **ST3164B/PBR​** | Met​ | wt | ESR1_CCDC170​ | ​ |  |
|  |  |  |  |  |  |
| **ST941/PBR/HI​** | Met​ | wt | Y537S​ | TP53m​ |  |
|  |  |  |  |  |  |

**Supplementary Table 13. Antibodies**

| Antibody (method) | Company | Catalogue Number |
| --- | --- | --- |
| Total RB | Cell Signalling Technologies (CST) | #9309 |
| Phospho RB | CST | #8516 |
| CDK4 | CST | #12790 |
| CDK6 | CST | #3136 |
| Cyclin D1 | Abcam | ab134175 |
| Cyclin E1 | CST | #4129 |
| E2F1 | CST | #3472 |
| p21 | CST | #2947 |
| Cyclin B1 | CST | #4138 |
| Cyclin A2 | CST | #91500 |
| RAD51 | CST | #8875 |
| Total AKT | CST | #9272 |
| Phospho AKT S473 | CST | #4060 |
| Total PRAS40 (in vitro) | CST | #2691 |
| Total PRAS40 (PD) | CST | #2610 |
| Phospho PRAS40 T246(in vitro) | CST | #2997 |
| Phospho PRAS40 T246 (PD) | CST | #13175 |
| Total S6 | CST | #2217 |
| Phospho S6 S235/236 (in vitro) | CST | #2211 |
| Phospho S6 S235/236 (PD) | CST | #4858 |
| Total 4E-BP1 | CST | #9452 |
| Phospho 4E-BP1 Thr37/46 | CST | #9459 |
| Phospho GSK3ᵝ | CST | #9336 |
| ERα | Thermo Fisher Scientific (TFS) | RM9101R |
| PR | Agilent Dako | PR636 |
| GREB-1 | CST | #65171 |
| c-Myc | CST | #5605 |
| PARP | CST | #9542 |
| Phospho Histone H2A.X S139 | Merck KGaA | 05-636 |
| GAPDH | CST | #2118 |
| B-actin | CST | #4970 |
| Vinculin (in vitro) | Sigma | V9131 |
| Vinculin (PD) | CST | #13901 |

**Supplementary Table 14. IHC Reagents**

| **Item** | **Source** |  |
| --- | --- | --- |
| **Ki67 (30-9) #790-4286** | Roche Diagnostics |  |
| **Antibody Block** | Roche Diagnostics |  |
| **Discovery OmniMap anti-Rb HRP** | Roche Diagnostics |  |
| **Discovery ChromoMap DAB Kit** | Roche Diagnostics |  |
| **Heamatoxylin II** | Roche Diagnostics |  |
| **Blueing Reagent** | Roche Diagnostics |  |
