# Supplementary material for: Combining the AKT inhibitor capivasertib and SERD fulvestrant is effective in palbociclib-resistant ER+ breast cancer preclinical models
Source: NPJ Breast Cancer. 2023 Aug 5;9:64. doi: 10.1038/s41523-023-00571-w (PMC10404292; doi:10.1038/s41523-023-00571-w)
Supplement: Supplementary file 2 — Supplementary Figure 1-7 [file 41523_2023_571_MOESM2_ESM.pptx]

## Slide 1
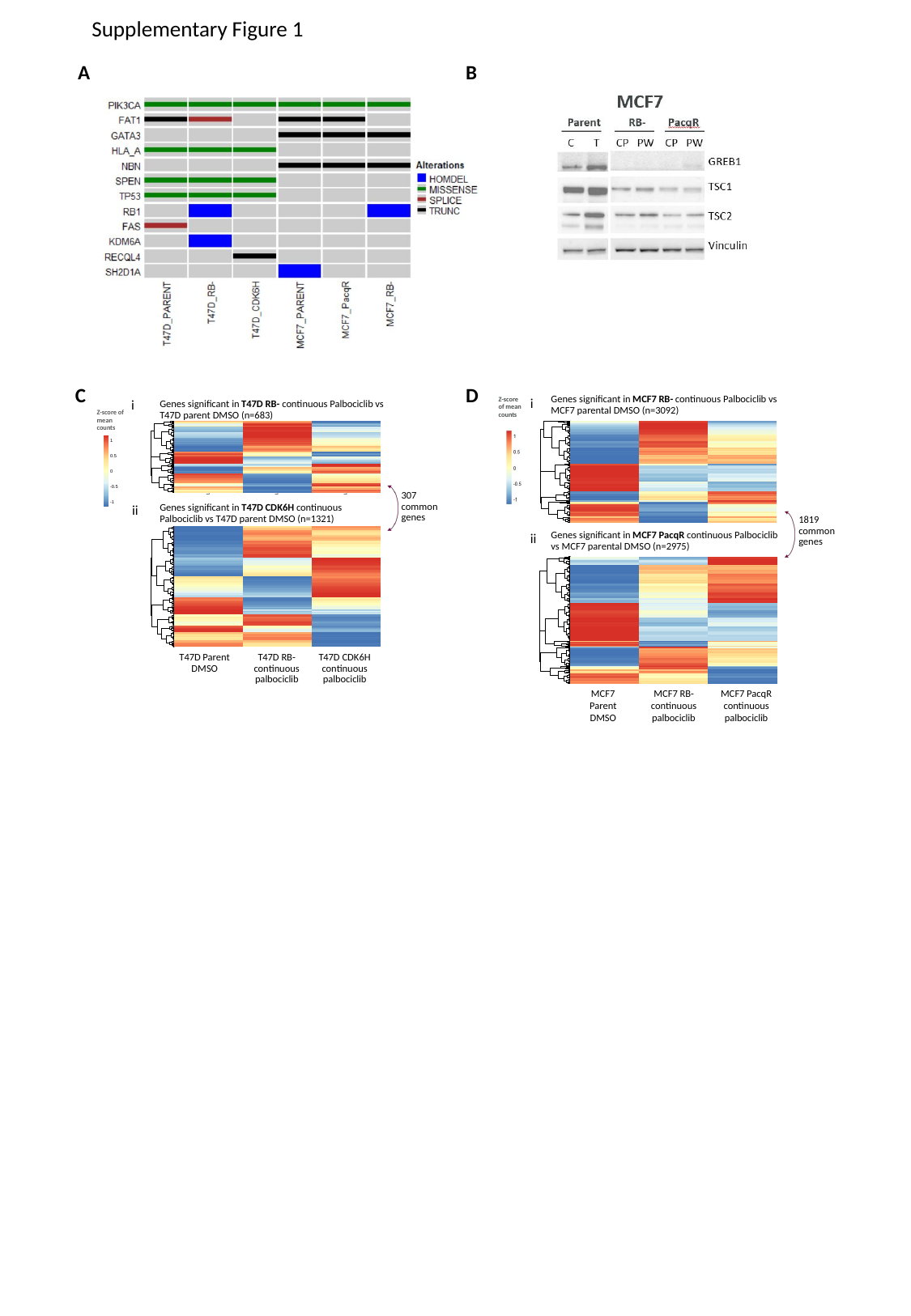

Supplementary Figure 1
A
B
C
D
Genes significant in MCF7 RB- continuous Palbociclib vs MCF7 parental DMSO (n=3092)
Z-score of mean counts
i
i
Genes significant in T47D RB- continuous Palbociclib vs T47D parent DMSO (n=683)
Z-score of mean counts
307 common genes
Genes significant in T47D CDK6H continuous Palbociclib vs T47D parent DMSO (n=1321)
ii
1819 common genes
ii
Genes significant in MCF7 PacqR continuous Palbociclib vs MCF7 parental DMSO (n=2975)
T47D Parent DMSO
T47D RB- continuous palbociclib
T47D CDK6H continuous palbociclib
MCF7 Parent DMSO
MCF7 RB- continuous palbociclib
MCF7 PacqR continuous palbociclib

## Slide 2
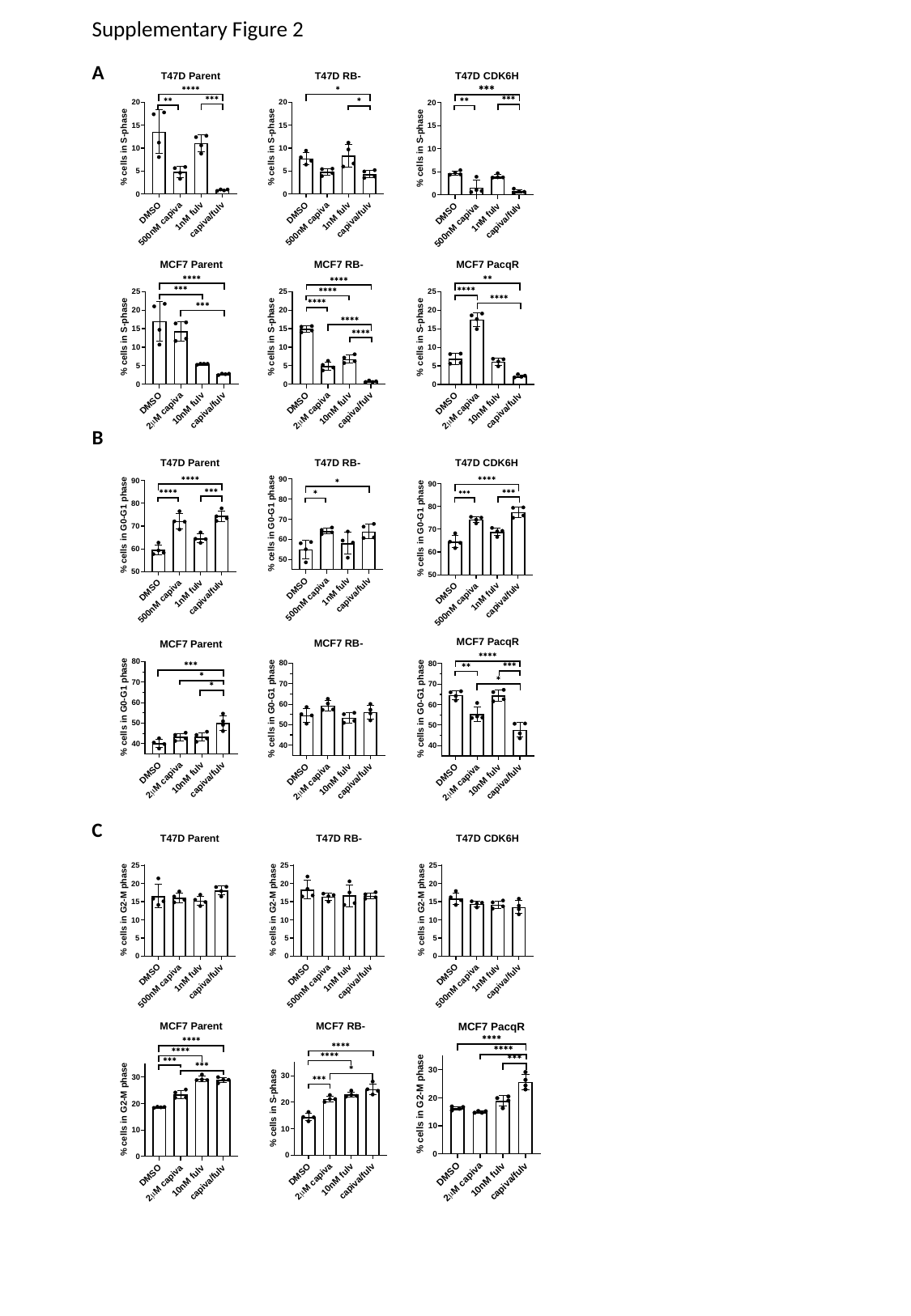

Supplementary Figure 2
A
B
C

## Slide 3
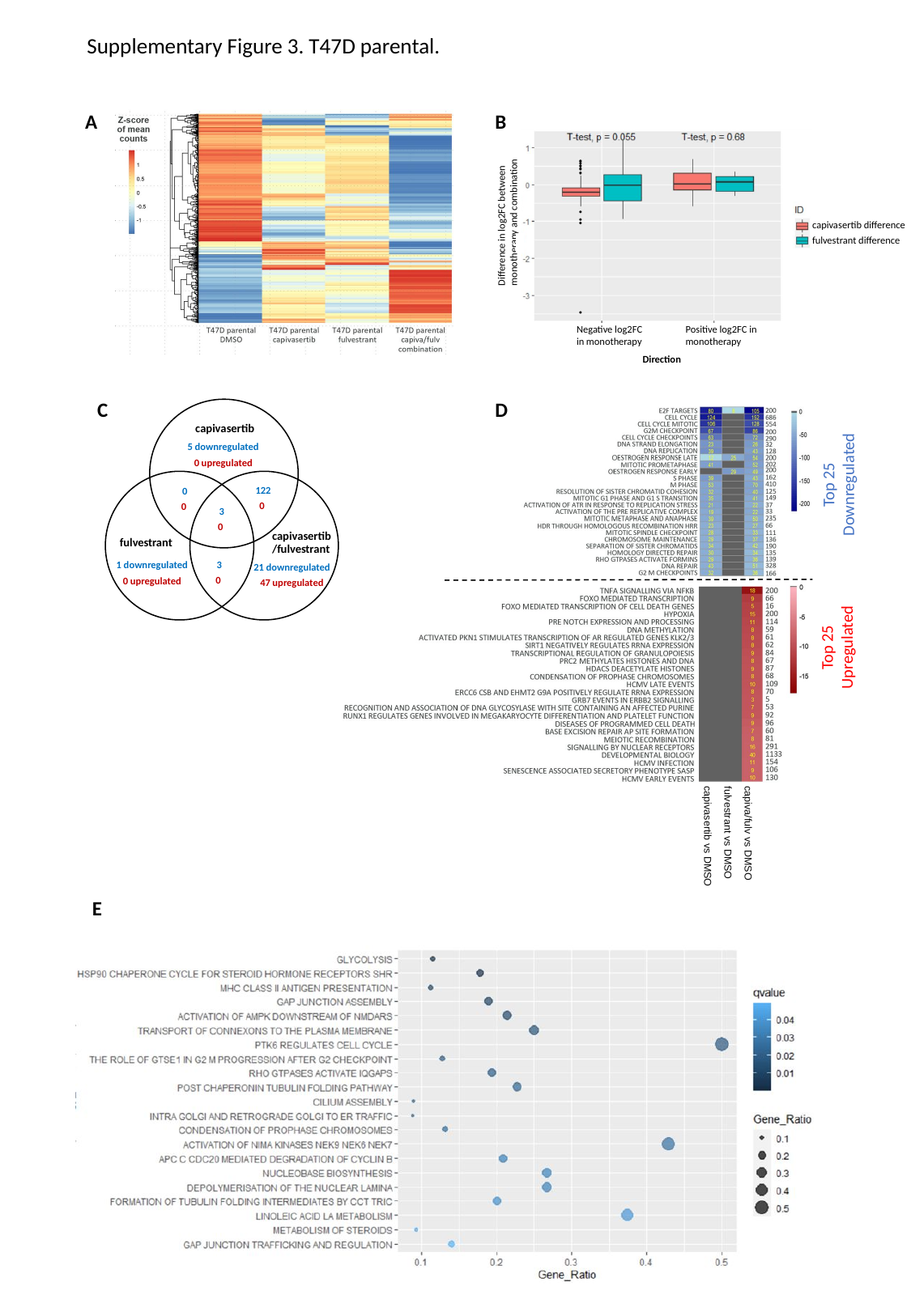

Supplementary Figure 3. T47D parental.
A
B
Difference in log2FC between monotherapy and combination
capivasertib difference
fulvestrant difference
Negative log2FC in monotherapy
Positive log2FC in monotherapy
Direction
D
C
capivasertib
5 downregulated
0 upregulated
122
0
0
0
3
0
capivasertib/fulvestrant
fulvestrant
3
0
1 downregulated
0 upregulated
21 downregulated
47 upregulated
Top 25 Downregulated
capiva/fulv vs DMSO
fulvestrant vs DMSO
capivasertib vs DMSO
Top 25 Upregulated
E

## Slide 4
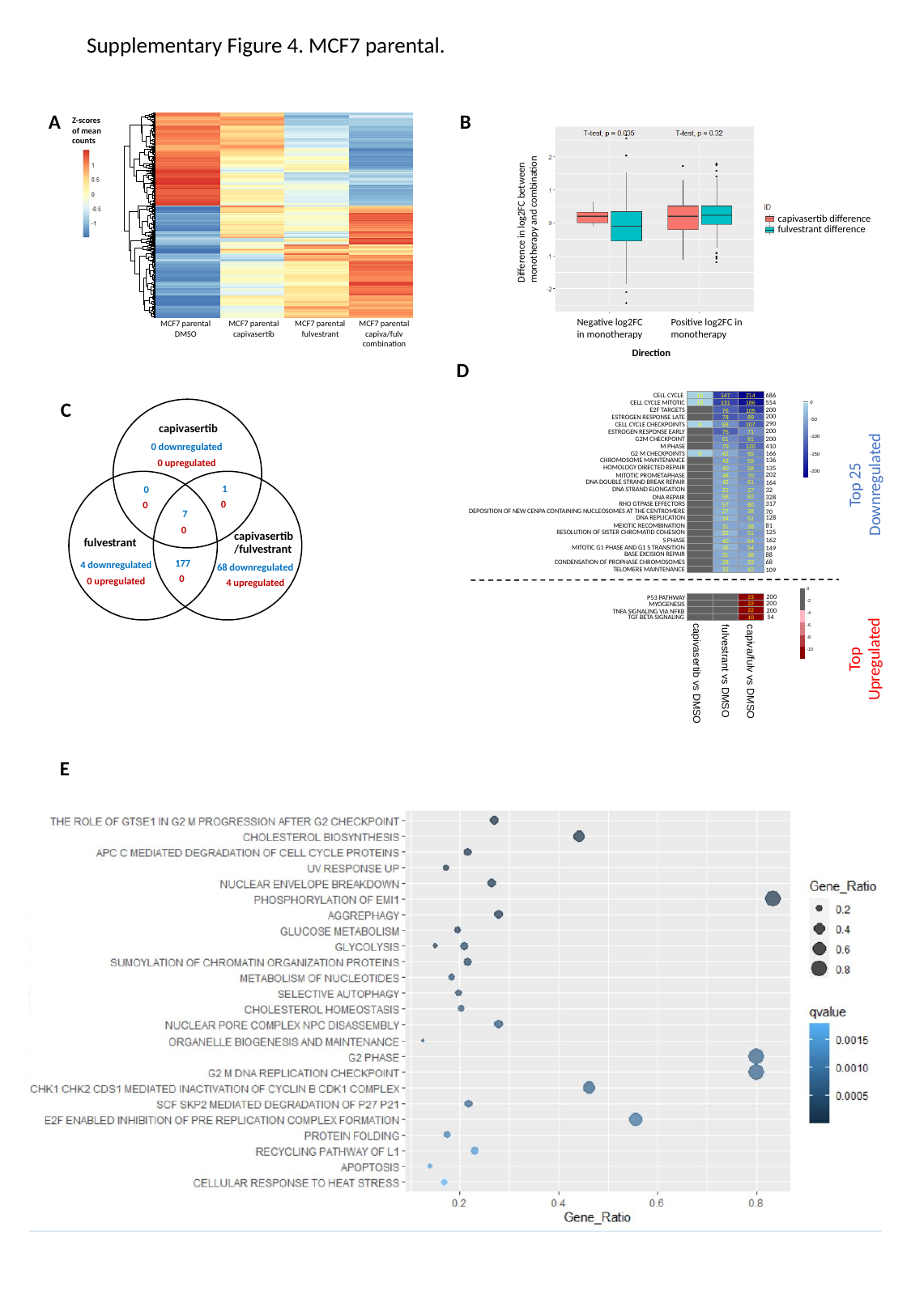

Supplementary Figure 4. MCF7 parental.
A
B
Z-scores of mean counts
Difference in log2FC between monotherapy and combination
capivasertib difference
fulvestrant difference
Negative log2FC in monotherapy
Positive log2FC in monotherapy
MCF7 parental DMSO
MCF7 parental capivasertib
MCF7 parental fulvestrant
MCF7 parental capiva/fulv combination
Direction
D
CELL CYCLE
686
C
554
CELL CYCLE MITOTIC
capivasertib
0 downregulated
0 upregulated
1
0
0
0
7
0
capivasertib/fulvestrant
fulvestrant
177
0
4 downregulated
0 upregulated
68 downregulated
4 upregulated
200
E2F TARGETS
200
ESTROGEN RESPONSE LATE
290
CELL CYCLE CHECKPOINTS
200
ESTROGEN RESPONSE EARLY
200
G2M CHECKPOINT
410
M PHASE
166
G2 M CHECKPOINTS
CHROMOSOME MAINTENANCE
136
HOMOLOGY DIRECTED REPAIR
135
Top 25 Downregulated
202
MITOTIC PROMETAPHASE
DNA DOUBLE STRAND BREAK REPAIR
164
DNA STRAND ELONGATION
32
DNA REPAIR
328
317
RHO GTPASE EFFECTORS
DEPOSITION OF NEW CENPA CONTAINING NUCLEOSOMES AT THE CENTROMERE
70
128
DNA REPLICATION
MEIOTIC RECOMBINATION
81
125
RESOLUTION OF SISTER CHROMATID COHESION
162
S PHASE
MITOTIC G1 PHASE AND G1 S TRANSITION
149
BASE EXCISION REPAIR
88
68
CONDENSATION OF PROPHASE CHROMOSOMES
TELOMERE MAINTENANCE
109
200
P53 PATHWAY
200
MYOGENESIS
200
54
TNFA SIGNALING VIA NFKB
TGF BETA SIGNALING
Top Upregulated
capivasertib vs DMSO
fulvestrant vs DMSO
capiva/fulv vs DMSO
E

## Slide 5
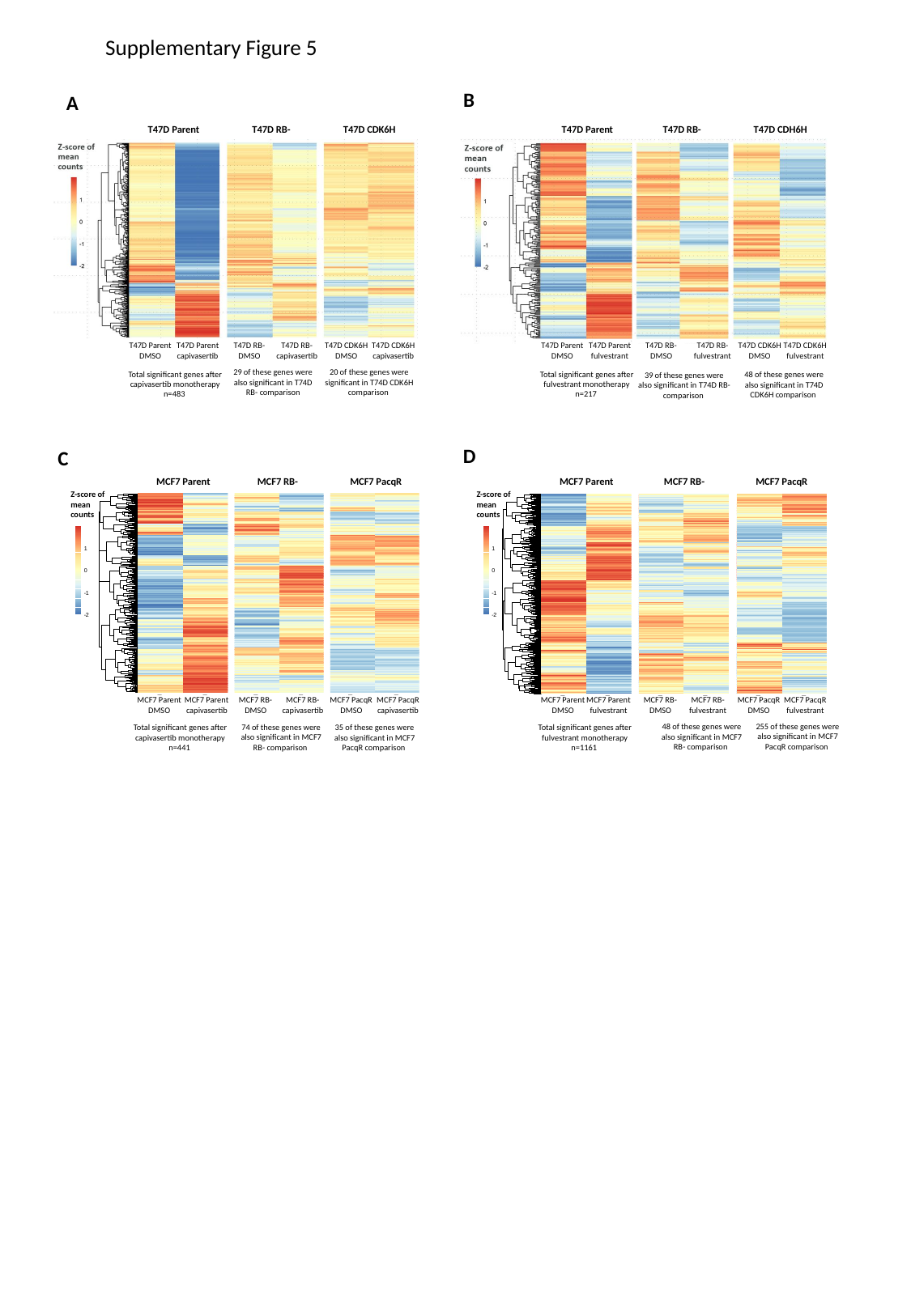

Supplementary Figure 5
B
A
T47D Parent
T47D RB-
T47D CDK6H
T47D Parent
T47D RB-
T47D CDH6H
T47D Parent DMSO
T47D Parent capivasertib
T47D RB- DMSO
T47D RB- capivasertib
T47D CDK6H DMSO
T47D CDK6H capivasertib
T47D Parent DMSO
T47D Parent fulvestrant
T47D RB- DMSO
T47D RB- fulvestrant
T47D CDK6H DMSO
T47D CDK6H fulvestrant
20 of these genes were significant in T74D CDK6H comparison
29 of these genes were also significant in T74D RB- comparison
Total significant genes after capivasertib monotherapy n=483
Total significant genes after fulvestrant monotherapy n=217
48 of these genes were also significant in T74D CDK6H comparison
39 of these genes were also significant in T74D RB- comparison
D
C
MCF7 Parent
MCF7 RB-
MCF7 PacqR
MCF7 Parent
MCF7 RB-
MCF7 PacqR
Z-score of mean counts
Z-score of mean counts
MCF7 Parent DMSO
MCF7 Parent capivasertib
MCF7 RB- DMSO
MCF7 RB- capivasertib
MCF7 PacqR DMSO
MCF7 PacqR capivasertib
MCF7 Parent DMSO
MCF7 Parent fulvestrant
MCF7 RB- DMSO
MCF7 RB- fulvestrant
MCF7 PacqR DMSO
MCF7 PacqR fulvestrant
255 of these genes were also significant in MCF7 PacqR comparison
48 of these genes were also significant in MCF7 RB- comparison
74 of these genes were also significant in MCF7 RB- comparison
35 of these genes were also significant in MCF7 PacqR comparison
Total significant genes after capivasertib monotherapy n=441
Total significant genes after fulvestrant monotherapy n=1161

## Slide 6
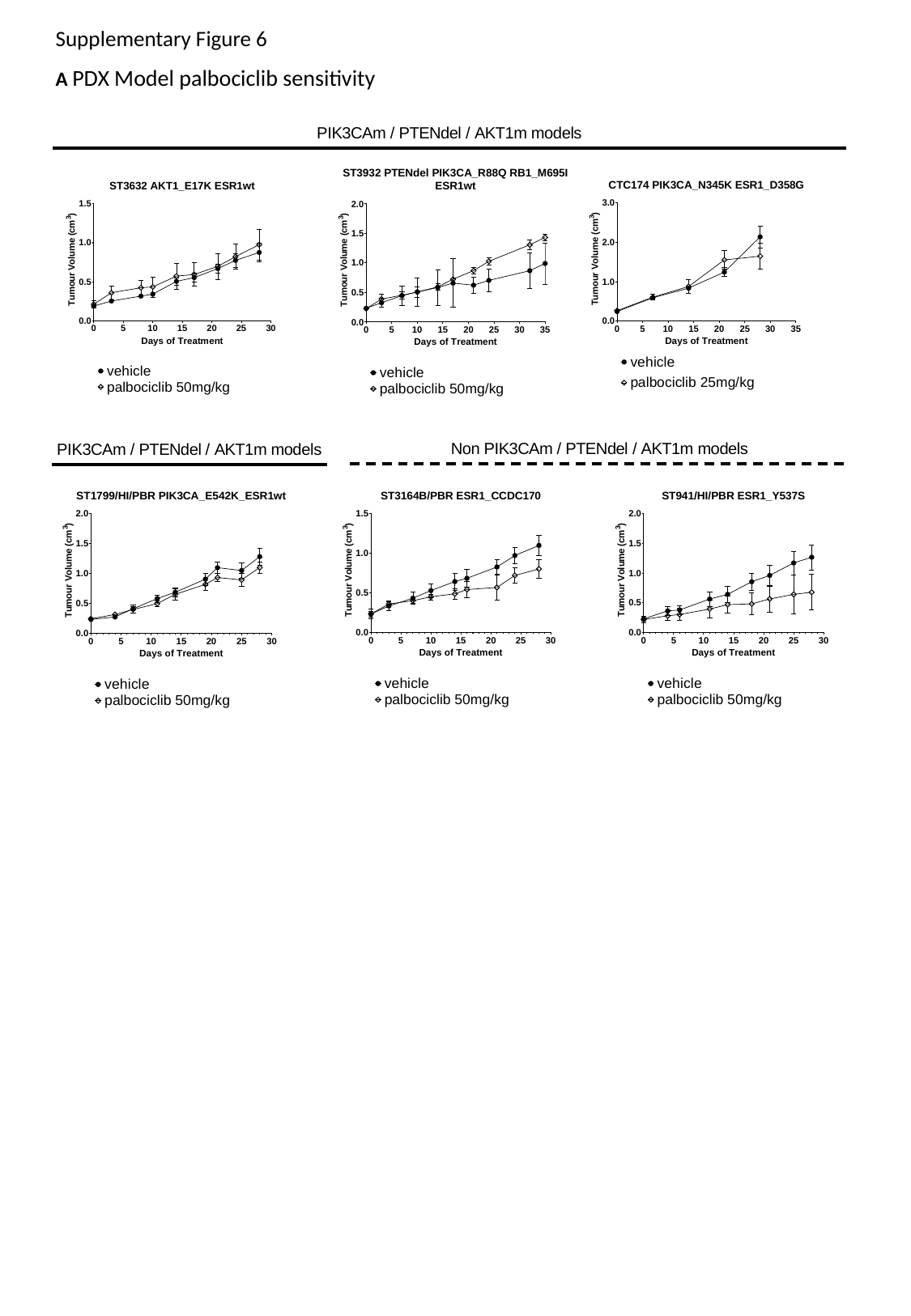

Supplementary Figure 6
A PDX Model palbociclib sensitivity

## Slide 7
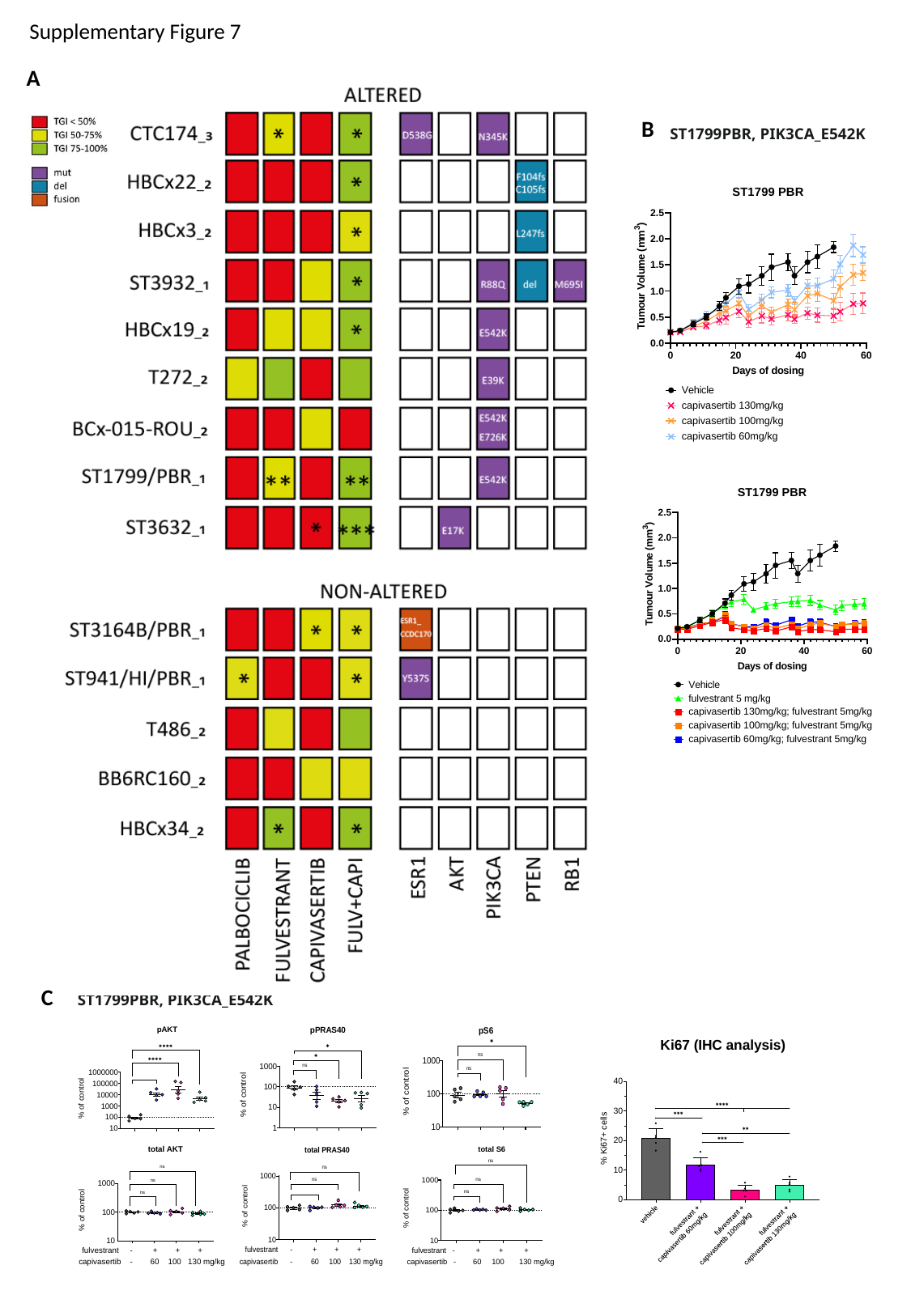

Supplementary Figure 7
A
B
ST1799PBR, PIK3CA_E542K
C
ST1799PBR, PIK3CA_E542K
